# Vegfc/d-dependent regulation of the lymphatic vasculature during cardiac regeneration is influenced by injury context

**DOI:** 10.1038/s41536-019-0079-2

**Published:** 2019-08-22

**Authors:** Céline J. Vivien, Cathy Pichol-Thievend, Choon Boon Sim, Jacob B. Smith, Neil I. Bower, Benjamin M. Hogan, James E. Hudson, Mathias Francois, Enzo R. Porrello

**Affiliations:** 1 Murdoch Children’s Research Institute, The Royal Children’s Hospital, Melbourne, VIC 3052 Australia; 2 Institute for Molecular Bioscience, The University of Queensland, Brisbane, QLD 4072 Australia; 3 School of Biomedical Sciences, The University of Queensland, Brisbane, QLD 4072 Australia; 4 QIMR Berghofer Medical Research Institute, Brisbane, QLD 4006 Australia; 5 Department of Physiology, School of Biomedical Sciences, The University of Melbourne, Melbourne, VIC 3010 Australia

**Keywords:** Angiogenesis, Cardiac regeneration

## Abstract

The lymphatic vasculature mediates essential physiological functions including fluid homeostasis, lipid and hormone transport, and immune cell trafficking. Recent studies have suggested that promoting lymphangiogenesis enhances cardiac repair following injury, but it is unknown whether lymphangiogenesis is required for cardiac regeneration. Here, we describe the anatomical distribution, regulation, and function of the cardiac lymphatic network in a highly regenerative zebrafish model system using transgenic reporter lines and loss-of-function approaches. We show that zebrafish lacking functional *vegfc* and *vegfd* signaling are devoid of a cardiac lymphatic network and display cardiac hypertrophy in the absence of injury, suggesting a role for these vessels in cardiac tissue homeostasis. Using two different cardiac injury models, we report a robust lymphangiogenic response following cryoinjury, but not following apical resection injury. Although the majority of mutants lacking functional *vegfc* and *vegfd* signaling were able to mount a full regenerative response even in the complete absence of a cardiac lymphatic vasculature, cardiac regeneration was severely impaired in a subset of mutants, which was associated with heightened pro-inflammatory cytokine signaling. These findings reveal a context-dependent requirement for the lymphatic vasculature during cardiac growth and regeneration.

## Introduction

In adults, lymphangiogenesis frequently occurs at sites of tissue injury and inflammation.^1–4^ This rapid lymphangiogenic response to injury is thought to contribute to the resolution of edema and clearance of immune cells at the wound site.^1,5–7^ Promoting lymphangiogenesis has therefore emerged as an attractive therapeutic strategy to facilitate tissue repair.^8–11^ However, whether the lymphangiogenic response influences endogenous tissue repair in highly regenerative organisms such as zebrafish is currently unclear. Identifying the cellular mechanisms and physiological processes that govern cardiac regeneration has the potential to inform new therapeutic approaches for ischemic heart disease and heart failure.

The heart relies on lymphatic vessels to maintain fluid balance, which must be tightly controlled to ensure normal cardiac output. The heart comprises an extensive lymphatic vascular plexus, which was originally thought to be entirely derived from the venous circuitry during development.^12,13^ However, a recent study has suggested that cardiac lymphatics may have a developmentally heterogeneous origin, whereby formation of part of the cardiac lymphatic network occurs independent of sprouting from the veins.^11^ The lymphatic vasculature has also emerged as a potential therapeutic target for ischemic heart disease with two independent studies reporting beneficial effects of recombinant vascular endothelial growth factor type C (VEGFC) delivery on cardiac function following experimental myocardial infarction (MI) in mice and rats.^10,11^ While these studies suggest that VEGFC delivery following MI is cardioprotective, the underlying cellular and molecular mechanisms are still poorly understood. Given that VEGFC has immunomodulatory functions that extend beyond its role on lymphatic endothelial cells (LECs),^14^ it is currently unclear whether the cardioprotective actions of VEGFC treatment following MI are due to promotion of VEGFC-dependent lymphangiogenesis.

VEGF family members are key mediators of blood vessel growth (angiogenesis) and lymphatic vessel growth (lymphangiogenesis). The VEGF family includes VEGFA, VEGFB, VEGFC, VEGFD, VEGFE, PLGF-1, and PLGF-2. VEFC and VEGFD are the most potent regulators of lymphangiogenesis. In mice and zebrafish, lymphatic vessel network assembly is dependent on VEGFR3 signaling through its ligand VEGFC.^6,15–17^ Heterozygous deletion of *Vegfr3* in mice causes severe lymphatic defects^18^ and mice lacking *Vegfc* fail to develop a lymphatic vasculature.^16^ In humans, patients harboring loss-of-function mutations in either *VEGFC* or *VEGFR3* develop lymphedema.^19^ Moreover, application of recombinant VEGFC provides a powerful stimulus for lymphangiogenesis in adults, which could be exploited in a number of therapeutic contexts including management of lymphedema and tissue repair.^20^ The other ligand for VEGFR3 is VEGFD, which is not required for development of the lymphatic vasculature in mice,^21^ but can compensate for loss of VEGFC in some contexts.^22^ VEGFD also functions at postnatal stages during the maturation of the lymphatic vascular network in the lung and dermal tissues.^23^ In zebrafish, *vegfd* is required for the development of facial lymphatics^24,25^ and is expressed throughout the embryonic trunk.^26^ Furthermore, genetic disruption of *vegfd* in a hypomorphic *vegfc* mutant background gives rise to severe lymphatic vascular defects in the trunk suggesting a redundant role for these two growth factors during lymphangiogenesis.^25^ In addition, *vegfd* is also required for artery hyperbranching during primary angiogenesis in zebrafish.^25^ Further, the genetic interaction of this growth factor with the transcription factor SOX18 has been shown to control artery caliber and angiogenic remodeling during mouse embryogenesis.^26^ Although VEGFA primarily drives angiogenesis via VEGFR2-dependent signaling, there is some evidence that VEGFA can also regulate lymphangiogenesis following inflammation.^27,28^ Due to their central roles as key mediators of blood vessel growth, VEGF family members are being pursued as therapeutic targets for a variety of disorders including cardiovascular disease.

To investigate the potential function of the lymphatic vasculature in cardiac homeostasis and tissue repair, we used *vegfc/d* loss-of-function in vivo to prevent lymphatic vascular remodeling and determine its impact on cardiac growth and endogenous regeneration following injury in zebrafish. Using a series of lymphatic reporter lines, we describe the major cardiac lymphatic vessels in adult zebrafish and use this system to analyze lymphangiogenesis following cardiac injury. Genetic deletion of *vegfc* and *vegfd* established a requirement for these signaling pathways for development of the cardiac lymphatic vasculature and for maintenance of heart size. Using two independent models of cardiac regeneration, we found a robust lymphangiogenic response occurred following cryoinjury, but not following apical resection injury. The regenerative response following cryoinjury was severely impaired in a subset of mutants lacking functional vegfc/d signaling, which was associated with an exacerbated inflammatory response. These findings reveal a surprising context-dependent requirement for the lymphatic vasculature during physiological cardiac growth and regeneration.

## Results

### Anatomical organization of the cardiac lymphatic network in adult zebrafish

**Fig. 1:**
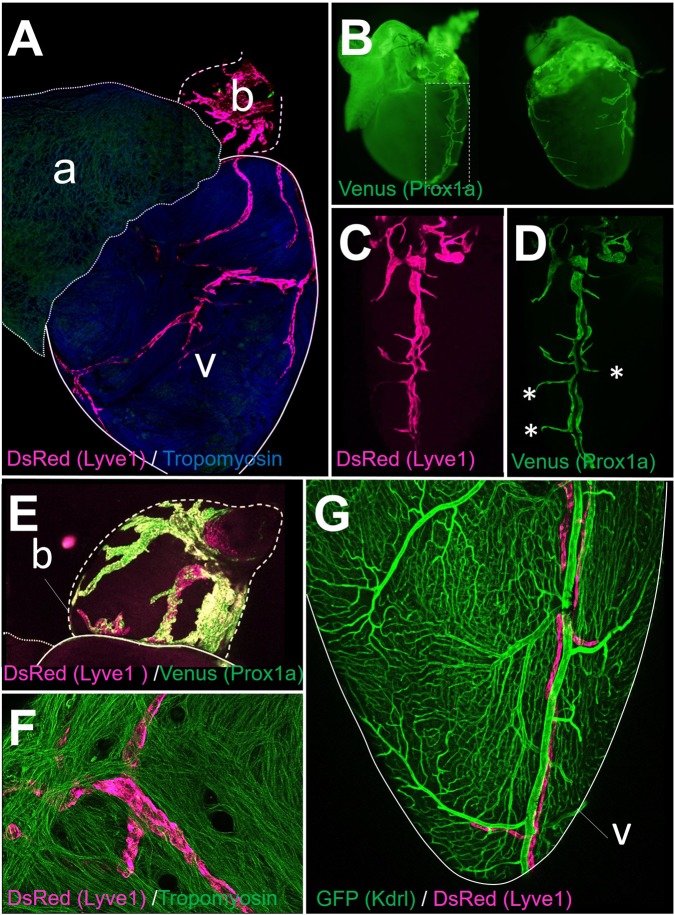
Anatomical organization of cardiac lymphatic vessels in adult zebrafish. **a** DsRed and Tropomyosin staining was performed on Tg(−5.2lyve1b:DsRed)^nz101^ adult zebrafish hearts. A dense lymphatic plexus was present in the bulbous (b) and a prominent lymphatic vascular network was detected on the ventricle (v). No lymphatics were detected on the atrium (a). **b** DsRed and Venus staining was performed on Tg(−5.2lyve1b:DsRed)^nz101^:Tg(prox1a:KalTA4)^uq3bh^ adult zebrafish hearts. Venus expression (Prox1a reporter) shows the same anatomical distribution of lymphatic vessels in the adult zebrafish heart compared with the Lyve1 reporter (**a**). High magnification images of the major lymphatic vessel and associated ramifications in the heart of Prox1 (**c**) and Lyve1 (**d**) reporter fish. There is a strong overlap in Lyve1 and Prox1 reporter expression, with the exception of a few small ramifications that were positive for Prox1 and negative for Lyve1 (*). **e** A broad network of large cardiac lymphatic vessels (DsRed and Venus stained) were detected on the bulbus arteriosus (b). **f** Ventricular lymphatics were predominantly located at the epicardial surface of the ventricle (confocal image taken from epicardial surface of the heart following whole-mount immunostaining for DsRed and Tropomyosin staining on Tg(−5.2lyve1b:DsRed)^nz101^ adult zebrafish heart). **g** Co-GFP and DsRed staining of Tg(kdrl:EGFP)^s834^:Tg(−5.2lyve1b:DsRed)^nz101^ adult zebrafish hearts. Lymphatic vessels (DsRed stained) are clearly distinct from blood vessels expressing Kdrl (GFP stained)

We firstly characterized the anatomical organization of the cardiac lymphatic vasculature in size-matched adult zebrafish using well established lymphatic reporter lines: Tg(−5.2lyve1b:DsRed)^nz101^ ^29^ and Tg(prox1a:KalTA4)^uq3bh^.^30^ Consistent with a LECs molecular identity, we observed a vessel network co-expressing both Prox1a and Lyve1 reporters in the adult zebrafish heart (Fig. 1). A highly-branched network of large lymphatic vessels was present on the bulbus arteriosus, while a less complex network of lymphatics harboring short ramifications was present on the cardiac ventricle (Fig. 1a–e). Ventricular lymphatics were primarily detected at the epicardial surface of the ventricle (Fig. 1a–c), but no vessels were detected on the atrium (Fig. 1a). To determine the proximity of the lymphatics and blood vessels of the heart, we used the Tg(kdrl:EGFP)^s834^:Tg(−5.2lyve1b:DsRed)^nz101^ reporter line,^29,31^ which allows for visualization of both blood vessels and lymphatics. As expected, the cardiac lymphatic network was clearly distinguishable from the coronary vasculature based on the visualization of this dual transgenic reporter line (Fig. 1g). The main lymphatic vessel in the cardiac ventricle ran parallel to the major coronary arteries in close proximity (Fig. 1g), but not all coronary vessels were surrounded by lymphatics (Fig. 1g). Together, these findings demonstrate that, in the absence of injury, the adult zebrafish heart contains a dense network of lymphatic vessels on the bulbous arteriosus and a prominent lymphatic vessel on the epicardial surface of the ventricle with few lymphatics penetrating deeper into the ventricular myocardium and no lymphatics within the atrium.

### Impairment of both vegfc and vegfd function prevents the formation of lymphatic vessels but does not block coronary vasculature assembly in the heart

**Fig. 2:**
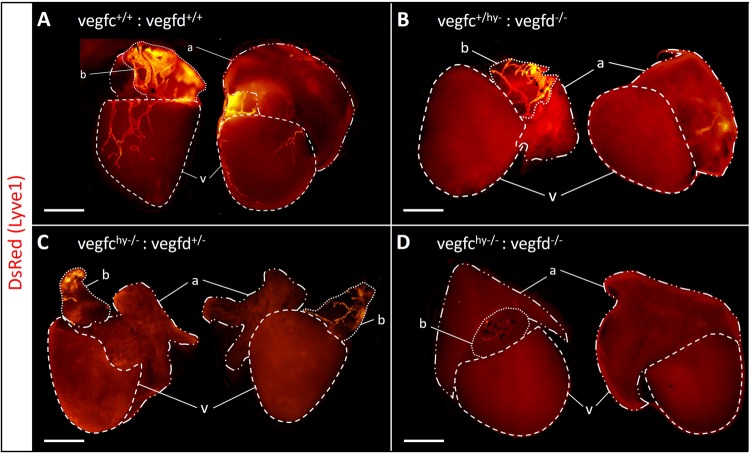
Vegfc and Vegfd are required for maintenance of the adult cardiac lymphatic vasculature. **a**–**d** Lymphatic reporter gene expression (Tg(−5.2lyve1b:Dred) in *vegfc* and *vegfd* mutant lines. There is no ventricular lymphatic network in *vegfc*^hy−/−^;*vegfd*^+/−^ and *vegfc*^+/hy−^:*vegfd*^−/−^ hearts (**b**, **c**). Also, there is an absence of lymphatic vascular network in both the ventricle and bulbus arteriosus of *vegfc*^hy−/−^;*vegfd*^−/−^ double mutants (**d**). Scale bar length: 1 mm

The formation of lymphatic vessels in zebrafish is highly dependent on the Vegfc/d-Vegfr3 signaling axis.^24,25,32,33^ Due to the early lethality of *vegfc* null mutants at day 7 postfertilization,^33^ we used hypomorphic *vegfc* mutants crossed to *vegfd* null mutants (vegfd^uq9bh^:vegfchu^5055^ double mutants hereto referred to as *vegfc*^hy−/−^;*vegfd*^−/−^), which survive to adulthood but have severely impaired lymphangiogenesis.^25^ In addition, *vegfc* and *vegfd* mutants were backcrossed into a Lyve1 transgenic reporter background (Tg(−5.2lyve1b:DsRed)^nz101^) to enable the visualization of the lymphatic vasculature. As expected, in control *vegfc*^*+*/+^;*vegfd*^*+*/+^:Tg(−5.2lyve1b:DsRed) fish DsRed expression was detected in lymphatic vessels within the bulbus arteriosus and within the cardiac ventricle, but was once again absent within the atrium (Fig. 2a). In *vegfc*^+/hy−^:*vegfd*^−/−^;Tg(−5.2lyve1b:DsRed) or *vegfc*^*hy*−/−^;*vegfd*^+/−^:Tg(−5.2lyve1b:DsRed) fish, lymphatics were absent around the ventricle, but were still detected in the bulbous arteriosus (Fig. 2b, c). This indicates that one functional allele of *vegfc* or *vegfd* alone is sufficient to establish and maintain the lymphatic vasculature in the bulbus arteriosus, which contains the highest density of lymphatic vessels in the zebrafish heart. In contrast, double mutants lacking both *vegfc* and *vegfd* alleles were almost completely devoid of lymphatic vessels on the bulbus arteriosus and did not comprise any lymphatics on the cardiac ventricle (Fig. 2d). There was no overt lethality in fish lacking functional *vegfc* and *vegfd*, indicating cardiac function at homeostasis was sufficient for survival without any requirement for a lymphatic vasculature in the heart. Importantly, cardiac blood vessels were detectable in the epicardium and myocardium of both *vegfc*^*+*/+^:*vegfd*^*+*/+^control and *vegfc*^*hy*−/−^;*vegfd*^−/−^mutant fish (Supplementary Fig. 1) indicating that loss of vegfc/d function did not overtly impact the development of the coronary vasculature in zebrafish.

### Dual loss-of-function of vegfc and vegfd causes cardiac hypertrophy in adult zebrafish

**Fig. 3:**
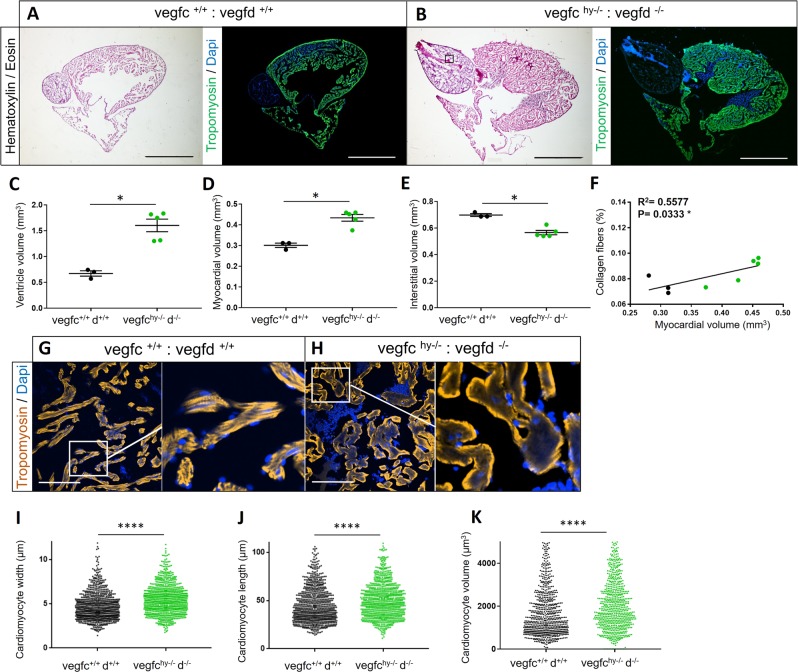
Loss of Vegfc/d signaling causes cardiac hypertrophy. Hematoxylin and Eosin or Tropomyosin and Dapi staining were performed on *vegfc*^*+*/+^:*vegfd*^*+*/+^ (**a**) and *vegfc*^−/−^:*vegfd*^hy−/−^ hearts (**b**). Scale bar length: 0.8 mm. Quantification of ventricle volume (**c**), myocardial volume (**d**), and interstitial volume (**e**) in *vegfc*^*+*/+^:*vegfd*^*+*/+^ and *vegfc*^hy−/−^:*vegfd*^−/−^ mutant hearts. There is marked cardiac hypertrophy with a reduced interstitial volume in *vegfc*^hy−/−^:*vegfd*^−/−^ double mutants. *n* = *3 vegfc*^*+*/+^:*vegfd*^*+*/+^ control hearts and 4 *vegfc*^hy−/−^:*vegfd*^−/−^ mutant hearts. Statistical analysis: unpaired, nonparametric, Mann–Whitney test was used to compare two means. Data are presented as the mean ± standard error of the mean (sem). **f** Correlation analysis between myocardial volume and collagen content in *vegfc*^*+*/+^*:vegfd*^*+*/+^ and *vegfc*^*hy*−*/*−^*:vegfd*^−*/*−^ mutant hearts. Black plots represent *vegfc*^*+*/+^*:vegfd*^*+*/+^ samples, green plots represent *vegfc*^*hy*−*/*−^*:vegfd*^−*/*−^ samples. There is a highly significant correlation between fibrosis and myocardial volume. **g**, **h** Tropomyosin and Dapi staining were performed on *vegfc*^*+*/+^:*vegfd*^*+*/+^ and *vegfc*^−/−^:*vegfd*^hy−/−^ hearts. Scale bar length: 100 μm. **i**–**k** Enzymatic digestion was performed on *vegfc*^*+*/+^*:vegfd*^*+*/+^ or *vegfc*^*hy*−*/*−^*:vegfd*^−*/*−^ mutant ventricles and isolated cardiomyocytes were measured. The black dots represents the *vegfc*^*+*/+^*:vegfd*^*+*/+^cardiomyocytes width (**i**), length (**j**), or volume (**k**) and the green dots represent *vegfc*^*hy*−*/*−^*:vegfd*^−*/*−^ mutant cardiomyocytes width (**i**), length (**j**), or volume (**k**). There are statistically significant differences in cardiomyocyte size between control and mutant hearts. Frequency distribution calculated from a total of ~200 cardiomyocytes per samples (~800 cardiomyocytes per group) comprising *n* = 4 *vegfc*^hy−/−^:*vegfd*^−/−^ ventricles and n = 4 *vegfc*^*+*/+^:*vegfd*^*+*/+^ ventricles, respectively. Statistical analysis: unpaired, nonparametric, Mann–Whitney test was used to compare the two groups. Significant differences are marked by an asterisk ****p < 0.0001

We next examined the hearts of *vegfc*^hy−/−^;*vegfd*^−/−^;Tg(−5.2lyve1b:DsRed) fish to identify any potential phenotypic outcome. Ventricular and myocardial volume were significantly increased by ~2.5-fold and ~1.5-fold, respectively, in *vegfc*^hy−/−^;*vegfd*^−/−^;Tg(−5.2lyve1b:DsRed) mutant hearts compared with controls, suggestive of a cardiac hypertrophic response (Fig. 3a–d). The hypertrophic phenotype was highly penetrant with 100% of the mutants having a lager ventricle volume (Fig. 3c) and increased myocardial volume (Fig. 3d) when compared with the controls. Interstitial volume was significantly reduced in *vegfc*^hy−/−^;*vegfd*^−/−^ hearts (Fig. 3e) due to an increased deposition of interstitial nonmuscular, non-collagenous tissue (Fig. 3a, b and Supplementary Fig. 2). There was no statistically significant difference in fibrosis between control and mutant hearts (Supplementary Fig. 2), although there was a highly significant correlation between fibrosis and the hypertrophic phenotype in the absence of injury (Fig. 3f). To determine whether the cardiac hypertrophic phenotype was due to an increase in cardiomyocyte size, we isolated cardiomyocytes by enzymatic digestion of control and mutant ventricles (Supplementary Fig. 2). Cardiomyocyte width, length, and volume were increased (Fig. 3g–k, Supplementary Fig. 2 and Supplementary Data Set 1) in *vegfc*^hy−/−^;*vegfd*^−/−^ mutant hearts consistent with hypertrophy of cardiomyocytes. These findings suggest that loss of vegfc/d function, which is required for the formation of lymphatic vessels in the heart, results in a highly penetrant cardiac hypertrophy phenotype.

### Transcriptomic profiling of v*egfc*^*hy*−/−^;*vegfd*^−/−^ mutant hearts reveals an upregulation of genes involved in cell proliferation and lipid metabolism

**Fig. 4:**
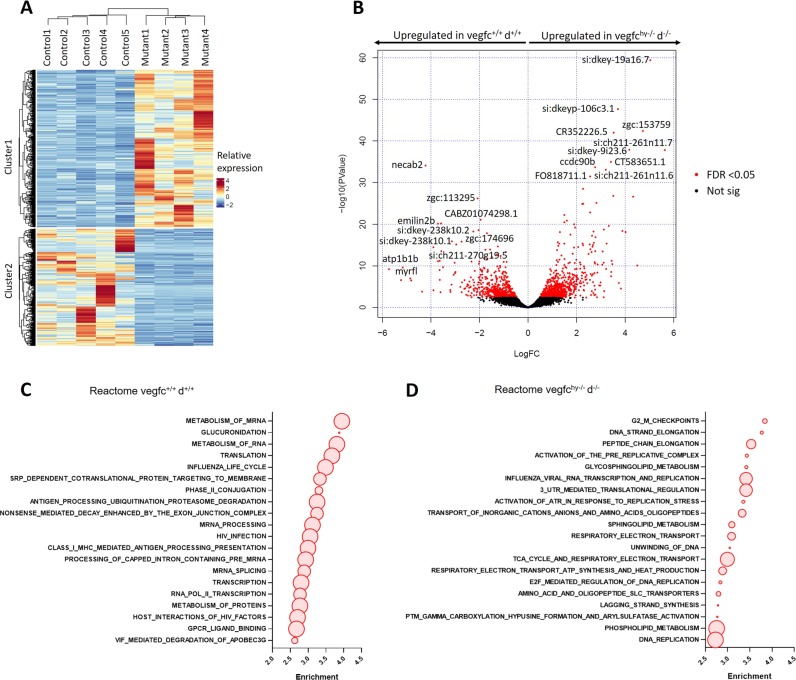
Transcriptomic profiling of v*egfc*^*hy*−/−^;*vegfd*^−/−^ mutant hearts reveals a disruption in cell proliferation and lipid metabolism. **a** Unsupervised hierarchical clustering showing distinct transcriptional profiles for *vegfc*^*+*/+^:*vegfd*^*+*/+^ control ventricles and in *vegfc*^hy−/−^:*vegfd*^−/−^ mutant ventricles. **b** Volcano plot of *vegfc*^*+*/+^:*vegfd*^*+*/+^ control ventricles and *vegfc*^hy−/−^:*vegfd*^−/−^ mutant ventricles. The top 10 upregulated genes for each comparison are indicated. Genes highlighted in red have a FDR < 0.05. Gene set enrichment analysis (GSEA) of *vegfc*^*+*/+^:*vegfd*^*+*/+^ control (**c**) and *vegfc*^*hy*−/−^;*vegfd*^−/−^ mutant ventricles (**d**). Representative pathways are highlighted with circles and gene set sizes are represented by circle sizes. GSEA indicates that the *vegfc*^*hy*−/−^;*vegfd*^−/−^ mutant ventricles are characterized by an upregulation of pathways related to cell proliferation and lipid metabolism compared with *vegfc*^*+*/+^:*vegfd*^*+*/+^ control hearts

To profile molecular pathways contributing to cardiac hypertrophy in *vegfc*^*hy*−/−^;*vegfd*^−/−^ mutants, we performed RNA-seq on cardiac ventricles from control and mutant zebrafish at 5 months of age (Fig. 4 and Supplementary Data Set 2). Unsupervised hierarchical clustering and principal component analysis (PCA) revealed distinct transcriptional signatures for control and mutant ventricles (Fig. 4a). In total, 616 genes with FDR < 0.05 were upregulated in control hearts and 828 genes were upregulated in mutant hearts (Fig. 4b). Gene set enrichment analysis (GSEA) revealed an enrichment for pathways related to mRNA processing and transcription in control ventricles (Fig. 4c), whereas mutant ventricles were characterized by an upregulation of pathways related to cell proliferation and lipid metabolism, in particular sphingolipid and phospholipid metabolism (Fig. 4d). Interestingly, v*egfc*^*hy*−/−^;*vegfd*^−/−^ mutant ventricles were not characterized by an upregulation of classic stress markers associated with pathological hypertrophy or hypertrophic cardiomyopathy in mammals (e.g., *nppa*, *nppb*, *myh7* or *acta1a*)^34^ (Supplementary Fig. 3). As cardiomyocyte hypertrophy is an uncommon phenotype in zebrafish and there are no established experimental models of cardiac hypertrophy in fish, we sought to determine the extent to which the transcriptional profile of v*egfc*^*hy*−/−^;*vegfd*^−/−^ mutant ventricles overlapped with the known molecular phenotype of established cardiac hypertrophy models in mice. To identify transcriptional networks that were common to the hypertrophic v*egfc*^*hy*−/−^;*vegfd*^−/−^ mutant zebrafish and established cardiac hypertrophy models, we crossed our data set with published data sets for both physiological and pathological cardiac hypertrophy induced by swim training and trans-thoracic aortic constriction (TAC) in mice, respectively (Supplementary Figs 3 and 4).^35^ GSEA revealed a number of transcriptional pathways that were shared between v*egfc*^*hy*−/−^;*vegfd*^−/−^ mutants and both physiological and pathological cardiac hypertrophy models^35^ (Supplementary Figs 3 and 4). Of note, cell cycle and lipid metabolism pathways were upregulated in both v*egfc*^*hy*−/−^;*vegfd*^−/−^ mutant zebrafish hearts and adult mouse hearts subjected to pressure overload following TAC (Supplementary Fig. 3). Conversely, genes involved in oxidative metabolism and the tricarboxylic acid (TCA) cycle were downregulated in both v*egfc*^*hy*−/−^;*vegfd*^−/−^ mutant zebrafish hearts and adult mouse hearts subjected to pressure overload following TAC. In contrast, pathways related to protein and RNA metabolism were shared between v*egfc*^*hy*−/−^;*vegfd*^−/−^ mutant zebrafish hearts and adult mouse hearts subjected to swim training (i.e., physiological hypertrophy) (Supplementary Fig. 4). Thus, some common features of the molecular cardiac hypertrophy program are shared between v*egfc*^*hy*−/−^;*vegfd*^−/−^ mutant zebrafish and established models of pathological and physiological cardiac hypertrophy in mice.

### Different types of injury cause distinct lymphangiogenic responses

**Fig. 5:**
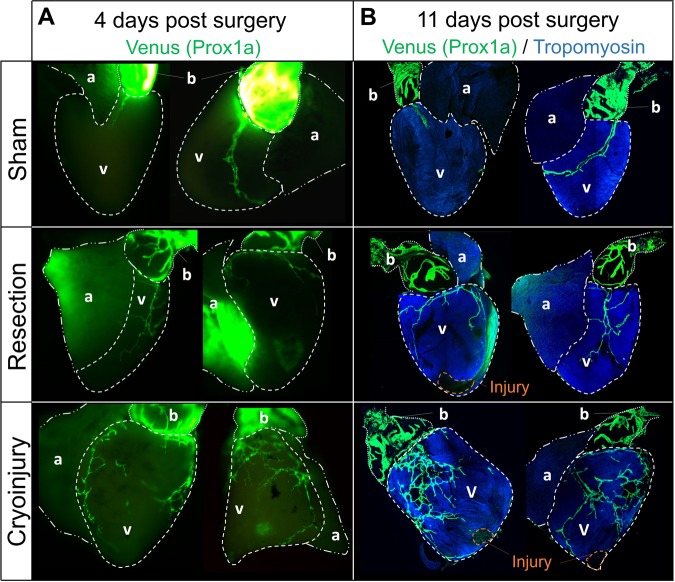
Cryoinjury stimulates a lymphangiogenic response in the adult zebrafish heart. **a** Whole-mount immunofluorescence staining of Tg(prox1a:KalTA4)^uq3bh^ adult zebrafish hearts at day 4 following sham surgery, apical resection, or cryoinjury. **b** Co-Venus and Tropomyosin staining was performed on Tg(prox1a:KalTA4)^uq3bh^ adult zebrafish hearts at day 11 following sham surgery, apical resection or cryoinjury. Note robust global lymphangiogenic response to cryoinjury compared with either apical resection or sham surgery

To test whether the cardiac lymphatic network is altered following cardiac injury, we subjected Tg(prox1a:KalTA4)^uq3bh^ lymphatic reporter fish to either apical resection or cryoinjury, both of which stimulate a robust cardiac regenerative response in zebrafish.^36–40^ To control for the impact of chest incision without myocardial damage on the cardiac lymphatic network, we performed sham surgeries and assessed the cardiac lymphatic response in the Tg(prox1a:KalTA4)^uq3bh^ reporter fish line. Following sham surgery, lymphatic vessels were detected on the bulbus arteriosus and the cardiac ventricle (Fig. 5), with a similar network coverage to wild-type control zebrafish (see Fig. 1), indicating that thoracic incision alone was insufficient to promote cardiac neo-lymphangiogenesis. We next assessed the cardiac lymphatics at 4 days or 11 days following apical resection injury. Apical resection injury did not stimulate overt cardiac lymphangiogenesis in adult zebrafish (Fig. 5). In contrast, global cardiac neo-lymphangiogenesis was clearly observed at 4 days and 11 days after cryoinjury, with the entire cardiac ventricle covered by neo-formed lymphatic vessels displaying an intricate network of ramifications (Fig. 5). Given this variable phenotypic response, we next wanted to assess whether this injury-specific response was also conserved in the regeneration of other organs. For these experiments we chose the fin, as both resection and cryoinjury also result in regeneration of this tissue.^41,42^ Consistent with the response of the heart, cryoinjury of the caudal fin stimulated an organ-wide activation of the lymphatic reporter transgene whereas amputation of this appendage failed to elicit any lymphatic vascular outgrowth (Supplementary Fig. 5). Overall, these results reveal that the lymphangiogenic response to tissue injury is highly model-dependent with cryoinjury providing a particularly powerful stimulus for neo-lymphangiogenesis.

### Context-dependent requirement for lymphangiogenesis for heart regeneration following cryoinjury

**Fig. 6:**
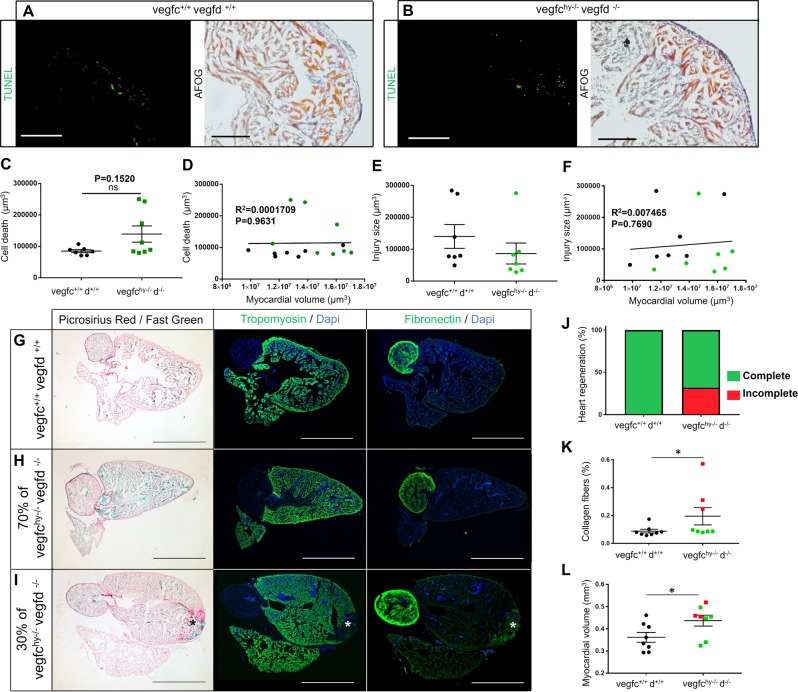
Vegfc/d-dependent lymphangiogenesis is dispensable for cardiac regeneration in adult zebrafish. TUNEL staining and AFOG staining were performed on *vegfc*^*+*/+^:*vegfd*^*+*/+^ (**a**) and *vegfc*^hy−/−^:*vegfd*^−/−^ hearts (**b**) at 1 day after cryoinjury. Scale bar length: 200 μm (TUNEL) or 100 μm (AFOG). **c** Quantification of cell death in *vegfc*^*+*/+^:*vegfd*^*+*/+^ and *vegfc*^hy−/−^:*vegfd*^−/−^ mutant hearts 1 day after cryoinjury. **d** Correlation analysis between myocardial volume and cell death in *vegfc*^*+*/+^*:vegfd*^*+*/+^ control and *vegfc*^*hy*−*/*−^*:vegfd*^−*/*−^ mutant hearts at 1 day post cryoinjury. Black plots represent *vegfc*^*+*/+^*:vegfd*^*+*/+^ samples, green plots represent *vegfc*^*hy*−*/*−^*:vegfd*^−*/*−^ samples. **e** Quantification of injury size in *vegfc*^*+*/+^:*vegfd*^*+*/+^ and *vegfc*^hy−/−^:*vegfd*^−/−^ mutant hearts at 1 day after cryoinjury. **f** Correlation analysis between myocardial volume and injury size in *vegfc*^*+*/+^*:vegfd*^*+*/+^ control and *vegfc*^*hy*−*/*−^*:vegfd*^−*/*−^ mutant hearts at 1 day post cryoinjury. Black plots represent *vegfc*^*+*/+^*:vegfd*^*+*/+^ samples, green plots represent *vegfc*^*hy*−*/*−^*:vegfd*^−*/*−^ samples. Statistical analysis: unpaired, nonparametric, Mann–Whitney test was used to compare two means. Data are presented as the mean ± standard error of the mean (sem), *n* = 8 *vegfc*^hy−/−^:*vegfd*^−/−^, *n* = 7 *vegfc*^*+*/+^:*vegfd*^*+*/+^. **g–i** First column: picrosirius red and fast green staining was performed on *vegfc*^*+*/+^:*vegfd*^*+*/+^ and *vegfc*^hy−/−^:*vegfd*^−/−^ hearts 6 months (180 days) after cryoinjury. Second column: tropomyosin staining was performed on *vegfc*^*+*/+^*:vegfd*^*+*/+^ and vegfc^hy−/−^:vegfd^−/−^ hearts 6 months after cryoinjury. Third column: fibronectin staining was performed on *vegfc*^*+*/+^:*vegfd*^*+*/+^ and *vegfc*^hy−/−^:*vegfd*^−/−^ hearts 6 months after cryoinjury. 30% of *vegfc*^hy−/−^:*vegfd*^−/−^ hearts display an impaired regenerative response with collagen deposition and fibrosis evident at the injury site (*). Scale bar length: 0.8 mm. **j** Percentage of hearts showing complete or incomplete cardiac regeneration at 180 days post cryoinjury. Quantification of collagen content (**k**) or myocardial volume (**l**) in *vegfc*^*+*/+^:*vegfd*^*+*/+^ and *vegfc*^hy−/−^:*vegfd*^−/−^ mutant hearts 6 months after cryoinjury. Black plots represent *vegfc*^*+*/+^*:vegfd*^*+*/+^ hearts, green plots represent *vegfc*^*hy*−*/*−^*:vegfd*^−*/*−^ hearts that showed complete heart regeneration, red plots represent *vegfc*^*hy*−*/*−^*:vegfd*^−*/*−^ hearts that had impaired cardiac regenerative capacity. Statistical analysis: unpaired, nonparametric, Mann–Whitney tests was used to compare two means. Data are presented as the mean ± standard error of the mean (sem), *n* = 8 *vegfc*^hy−/−^:*vegfd*^−/−^, *n* = 8 *vegfc*^*+*/+^:*vegfd*^*+*/+^. Significant differences are marked by an asterisk (*p < 0.05)

Compared with apex resection, cryoinjury is a more relevant pathophysiological model of infarction because the damaged tissue requires clearance and subsequent repair instead of a regrowth-based mechanism. To test whether lymphangiogenesis is required for cardiac regeneration, we subjected adult fish devoid of cardiac lymphatics (*vegfc*^hy/−^;*vegfd*^−/−^ double mutants) to cryoinjury, as this infarction model stimulated a reproducible and quantifiable lymphangiogenic response (Fig. 5). Hearts of both *vegfc*^*+*/+^:*vegfd*^*+*/+^ control fish or *vegfc*^*hy*−/−^;*vegfd*^−/−^ mutant fish were collected and analyzed at 1 day after cryoinjury to monitor the degree of cardiac damage (Fig. 6a–f). Terminal deoxynucleotidyl transferase dUTP nick end labeling (TUNEL) and Acid Fucshin Orange G (AFOG) staining revealed comparable infarct sizes between control and mutant hearts following cryoinjury (Fig. 6a–f). However, we noted that a subset of mutant hearts (3/8) displayed an accumulation of TUNEL-positive apoptotic/necrotic cells despite having similar infarct sizes (Fig. 6c and Supplementary Fig. 6), suggesting that a subset of *vegfc*^*hy*−/−^;*vegfd*^−/−^ mutants have an impaired capacity to clear dead cells following cryoinjury. Cell death and injury size were not correlated with myocardial volume (Fig. 6d, f), indicating that the degree of cardiac tissue damage was not influenced by heart size.

To determine whether lymphangiogenesis is required for cardiac regeneration, *vegfc*^hy/−^;*vegfd*^−/−^ double mutants and control hearts were collected and analyzed at 180 days after cryoinjury, which corresponds to a timepoint when the regenerative response is normally completed in wild-type zebrafish.^38–40,43^ Surprisingly, the vast majority (~70%) of *vegfc*^hy−/−^;*vegfd*^−/−^ mutants, which completely lack cardiac lymphatics, were able to mount a complete regenerative response without any signs of fibrosis (Fig. 6g–k and Supplementary Fig. 8). We found that the cardiac regenerative response was impaired only in a subset (3/8) of *vegfc*^hy−/−^;*vegfd*^−/−^ mutants, which were characterized by large deposits of fibrotic scar tissue present at the injury site (Fig. 6i-I”). Given that cardiomyocyte proliferation is required for zebrafish heart regeneration, we assessed *vegfc*^hy−/−^;*vegfd*^−/−^ mutant hearts at both early (day 19) and late (day 180) timepoints following cryoinjury to determine whether there was a defect in proliferation. Cardiomyocyte proliferation rates were unaffected at either timepoint in *vegfc*^hy−/−^;*vegfd*^−/−^ mutant hearts (Supplementary Figs. 8 and 9), including the 30% of mutants that had an impaired regenerative response (Supplementary Fig. 8). Conversely, in the subset of mutant hearts that displayed an impaired regenerative response, large infarcts and severe hypertrophy were evident at early and late timepoints following injury (Fig. 6k, l, and Supplementary Figs 8 and 9). Thus, vegfc/d signaling and lymphangiogenesis are not required for cardiomyocyte proliferation but appear to be required for efficient cellular clearance and scar resolution in a subset of zebrafish following cryoinjury.

### Transcriptomic profiling of non-regenerative v*egfc*^*hy*−/−^;*vegfd*^−/−^ mutant hearts reveals a marked inflammatory response

**Fig. 7:**
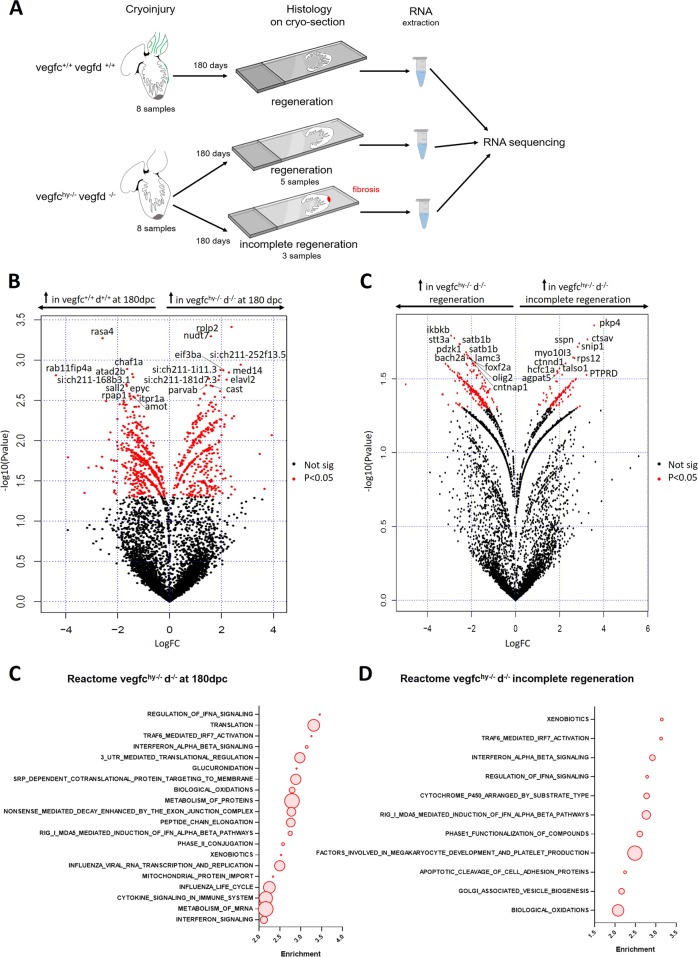
Transcriptomic profiling of v*egfc*^*hy*−/−^;*vegfd*^−/−^ mutant hearts at 180 days following cryoinjury. **a** Schematic representation of experimental design for RNA sequencing on cryosections. RNA-seq was performed on cryosections from control and mutant ventricles at 180 days following cryoinjury to compare transcriptional profiles to regenerative outcomes for the samples presented in Fig. 6g–l. **b** Volcano plot showing genes enriched in *vegfc*^*+*/+^:*vegfd*^*+*/+^ control ventricles at 180 days post cryoinjury and in *vegfc*^hy−/−^:*vegfd*^−/−^ mutant ventricles at 180 days after cryoinjury. The top 10 upregulated genes are indicated for each comparison. Genes highlighted in red have a *p*-value < 0.05. **c** Volcano plot showing genes enriched in *vegfc*^hy−/−^:*vegfd*^−/−^ mutant ventricles that did not regenerate compared with *vegfc*^hy−/−^:*vegfd*^−/−^ mutant ventricles that did regenerate at 180 days after cryoinjury. The top 10 upregulated genes are indicated for each comparison. Genes highlighted in red have a *p*-value < 0.05. **d** GSEA for *vegfc*^hy−/−^:*vegfd*^−/−^ mutant ventricles at 180 days after cryoinjury compared with *vegfc*^*+*/+^:*vegfd*^*+*/+^ control ventricles at 180 days post cryoinjury. Representative pathways are highlighted with circles and gene set sizes are represented by circle sizes. **e** GSEA for *vegfc*^hy−/−^:*vegfd*^−/−^ mutant ventricles that did not regenerate compared with *vegfc*^hy−/−^:*vegfd*^−/−^ mutant ventricles that did regenerate at 180 days post cryoinjury. Representative pathways are highlighted with circles and gene set sizes are represented by circle sizes. These data indicate that *vegfc*^*hy*−/−^;*vegfd*^−/−^ mutant ventricles are characterized by an upregulation of pathways related to inflammation, cytokine signaling and immune response compared with *vegfc*^*+*/+^:*vegfd*^*+*/+^ control hearts at 180 days post cryoinjury

To profile vegfc/d-dependent genetic pathways contributing to lymphangiogenesis in cardiac regeneration, we performed RNA-seq on cryosections from control and mutant ventricles at 180 days following cryoinjury. This analysis enabled us to directly compare transcriptional profiles with regenerative outcomes for the samples presented in Fig. 6g–l (Fig. 7a). Overall, *vegfc*^*hy*−/−^;*vegfd*^−/−^ mutant ventricles were characterized by an upregulation of pathways related to RNA and protein metabolism, cytokine signaling, and inflammation (Fig. 7c and Supplementary Data Set 3). In order to identify which pathways were specifically associated with injury in the mutant hearts, we cross-referenced these data to the baseline RNA-seq data set obtained from *vegfc*^*hy*−/−^;*vegfd*^−/−^ mutant hearts without injury (presented in Fig. 4) and then repeated the GSEA (Supplementary Fig. 10). Pathways related to cytokine signaling, innate immune system and inflammation were uniquely enriched after injury in the *vegfc*^*hy*−/−^;*vegfd*^−/−^ mutant hearts (Supplementary Fig. 10).

To determine the molecular basis of the regenerative capacity within the mutant fish population, we next compared the transcriptional profiles of *vegfc*^*hy*−/−^;*vegfd*^−/−^ mutant hearts that did not regenerate (*n* = 3) with the profiles of mutant hearts that recovered completely (*n* = 5) at 180 days post cryoinjury (presented in Fig. 6g–l). The subset of *vegfc*^*hy*−/−^;*vegfd*^−/−^ mutant hearts that failed to regenerate were characterized by an enrichment of inflammatory pathways, in particular TRAF6-mediated IRF7 activation, interferon alpha beta signaling and regulation of IFNA signaling (Fig. 7d, and Supplementary Data Set 4). These findings indicate that loss of the vegfc/d signaling axis is associated with a sustained and pronounced inflammatory response following injury. This change in the cardiac micro-environment seems to favor adverse conditions, which impair the regenerative process.

## Discussion

Here, we characterize the lymphatic vasculature of the adult zebrafish heart and assess its potential role in cardiac regeneration. Analysis of the lymphatic vasculature revealed a prominent cardiac lymphatic vessel network on the epicardial surface of the heart, which included a major lymphatic vessel located in close proximity to the main coronary artery and numerous branching vessels. In contrast, lymphatics were not detected in the atrium. The anatomical organization of the cardiac lymphatic vasculature in zebrafish is similar to the mammalian heart, which comprises a greater density of lymphatic vessels on the ventricles compared with the atria.^44^ Interestingly, the fish heart is also characterized by a dense lymphatic network in the bulbus arteriosus, which could reflect the specialized physiological function of this anatomical structure in the fish circulatory system. The bulbus arteriosus has a thick fibro-elastic wall and functions as a capacitor to normalize the ventricular pulse pressure as blood exits the ventricle and is pumped out to the gills.^45^ Thus, the presence of lymphatic vessels in the zebrafish heart is correlated with blood pressure in the different chambers, where the chambers under the highest pressure have the greatest density of lymphatic vessels. This likely reflects the central role of the lymphatic system in fluid homeostasis.

Our findings indicate that the development of the cardiac lymphatic vasculature is dependent on VEGFC and VEGFD signaling. Genetic loss-of-function of either *vegfc* or *vegfd* was sufficient to abolish the development of the ventricular lymphatic network in a gene dosage dependent manner. In contrast, a single copy of either *vegfc* or *vegfd* was sufficient to maintain the lymphatic vascular network on the bulbus arteriosus, which was only abolished when both *vegfc* and *vegfd* function were compromised. However, it should be noted that a few lymphatic vessels could still be detected on the bulbous arteriosus of *vegfc*^hy−/−^;*vegfd*^−/−^ double mutants. The presence of a few lyve1-expressing cells on the bulbus arteriosus of double mutant fish might reflect the existence of an alternative Vegfc/d-independent pathway for lymphangiogenesis in the heart or, alternatively, could be indicative of residual Vegfc signaling in this hypomorphic mutant.^32^ Nevertheless, *vegfc*^hy−/−^:*vegfd*^−/−^ double mutants completely lacked a ventricular lymphatic network, which did not affect organismal viability. Importantly, there was also no effect on the development of the coronary vasculature, which enabled the specificity of the lymphatic network to be studied.

A robust lymphangiogenic response occurred following cryoinjury, but not following resection injury in the heart and caudal fin. It is important to note that a regenerative response is observed in the heart and caudal fin following either resection or cryoinjury despite the apparent discrepancy in lymphangiogenic responses to different types of tissue damage.^36–38,40–43^ Surgical amputation (resection) and cryoinjury models differ with respect to the size of the injury, presence of cellular debris following tissue damage, and the degree of inflammation induced after injury, which are all more pronounced following cryoinjury.^36–38,40,43,46^ Interestingly, macrophages can secrete VEGF-C to promote lymphangiogenesis, which could provide a potential link between inflammation and lymphangiogenesis depending on injury context. While the precise mechanisms accounting for differences in the lymphatic response to apical resection versus cryoinjury require further investigation, it is likely that the degree of inflammation is a key regulator that dictates the nature of the lymphangiogenic response.

We were surprised to find that the majority of adult zebrafish were still capable of mounting a complete regenerative response following cryoinjury even in the absence of a functional cardiac lymphatic network. Therefore, the cardiac lymphatic vasculature appears to be dispensable for cardiomyocyte proliferation and clearance of fibrotic scar tissue in the majority of regenerating hearts. However, cardiac regeneration was severely impaired in a subset of mutants, which were characterized by fibrotic scars, severe cardiac hypertrophy, and a prominent inflammatory signature up to 180 days following cryoinjury. This variable response to cryoinjury in a subset of mutants was apparent as early as 1 or 19 days following cryoinjury, where some mutants were characterized by an accumulation of apoptotic/necrotic cells at day 1 and larger infarcts at day 19. This phenotype is consistent with the known role of lymphatics in the clearance of cellular debris following injury.^47^ In addition, non-regenerative mutant hearts displayed an enrichment in inflammatory pathways associated with TRAF6-mediated IRF7 activation and interferon alpha/beta signaling, which are involved in immune cell activation, recruitment, and clearance.^48,49^ In the absence of a functional lymphatic network, *vegfc*^hy−/−^:*vegfd*^−/−^ mutant hearts may have an impaired capacity to resolve inflammation following injury, which could impair the regenerative process when inflammation is heightened under certain conditions. Intrinsic differences in the susceptibility to myocardial injury and inflammation could be related to differences in genetic background, which is known to influence infarct size in mice.^50^ Together, these findings suggest that Vegfc/d-dependent lymphangiogenesis may facilitate cardiac regeneration under certain circumstances, for example, when there is a heightened inflammatory response to injury.

**Fig. 8:**
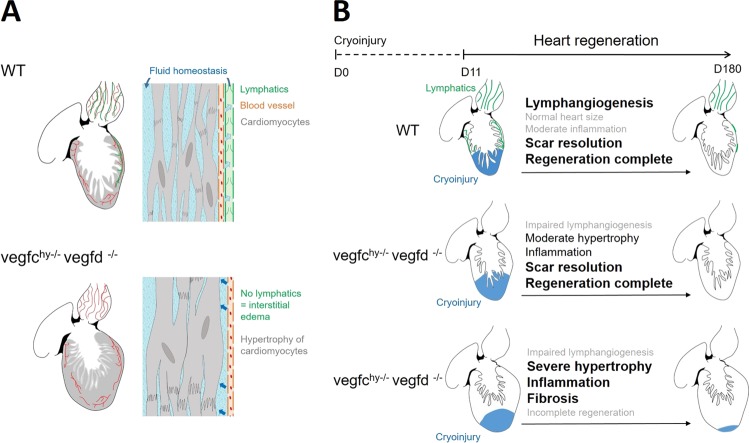
Proposed model showing relationship between lymphangiogenesis, cardiac hypertrophy and cardiac regeneration. **a** In wild-type (WT) zebrafish, the cardiac lymphatic network (shown in green) is present on the bulbus arteriosus and on the ventricle but is not detected on the atrium. Genetic deletion of either *vegfc* or *vegfd* is sufficient to abolish the development of the cardiac lymphatic network and induces a marked increase in myocardial wall volume, ventricular enlargement, hypertrophy of cardiomyocytes and a decrease in myocardial interstitial volume. The absence of lymphatics can cause interstitial edema because of the impaired regulation of fluid balance. Blood vessels (shown in red) are not affected by vegfc/vegfd and are present in both *vegfc*^*+*/+^*:vegfd*^*+*/+^ control hearts and *vegfc*^*hy*−*/*−^*:vegfd*^−*/*−^ mutants hearts. **b** A robust lymphangiogenic response is induced by cryoinjury in WT fish. The majority of *vegfc*^*hy*−*/*−^*:vegfd*^−*/*−^ mutants display moderate hypertrophy and are able to fully regenerate their heart even in the absence of a functional cardiac lymphatic vascular network and moderate inflammation. However, a subset of mutants display an incomplete cardiac regenerative response following cryoinjury. A heightened and persistent inflammatory response to injury could in part explain why a subset of mutants have an impaired regenerative response and severe cardiac hypertrophy following cryoinjury

The most striking cardiac phenotype observed in zebrafish lacking Vegfc and Vegfd was a marked increase in myocardial wall volume, ventricular enlargement, and hypertrophy of cardiomyocytes. Several potential mechanisms could contribute to cardiac hypertrophy in *vegfc*^hy−/−^:*vegfd*^−/−^ mutants. Hypertrophy of mutant cardiomyocytes is consistent with increased myocardial wall stress due to pressure overload,^51^ which would be expected if there is interstitial edema in mutant hearts lacking a functional lymphatic system (Fig. 8). Interestingly, classic molecular markers of pathological cardiac hypertrophy such as *nppa*, *nppb*, *myh7*, and *acta1a* were not upregulated in mutant zebrafish hearts.^34^ However there was some overlap between transcriptional pathways in the v*egfc*^*hy*−/−^;*vegfd*^−/−^ mutant ventricles and the molecular programs associated with established experimental models of physiological and pathological cardiac hypertrophy in mice.^35^ In particular, pathways related to cell proliferation and lipid metabolism were enriched in mutant zebrafish hearts and mouse hearts subjected to pressure overload. The enrichment of transcripts related to cell proliferation likely reflects an infiltration of proliferating non-myocytes rather than cardiomyocyte proliferation, as cardiomyocyte proliferation indices were not different between mutant and control ventricles and cell cycle-associated transcripts are typically enriched in non-myocyte populations in the adult mouse heart following injury.^52^ Although the precise mechanism driving cardiac hypertrophy in *vegfc*^hy−/−^:*vegfd*^−/−^ mutants is unclear, a striking enrichment of genes implicated in sphingolipid and phospholipid metabolism was observed. The lymphatic vasculature is known to play an important role in lipid transport.^53^ It is currently unclear how dysregulated lipid metabolism might contribute to cardiac hypertrophy, but a recent study reported that physiological and pathological cardiac hypertrophy are characterized by distinct lipid profiles including enrichment of several sphingolipid and phospholipid species.^54^ Sphingolipid metabolites are also important regulators of inflammation and immune cell trafficking,^55^ which could be linked to the heightened expression of pathways related to inflammation and cytokine signaling in *vegfc*^*hy*−/−^;*vegfd*^−/−^ mutant zebrafish following cryoinjury. Further studies are required to dissect the role of sphingolipid and phospholipid metabolism in cardiac hypertrophy and inflammation.

A recent study also reported that *vegfaa* over-expression results in changes in cardiac energy utilization in zebrafish, suggesting that VEGF family members are important regulators of myocardial metabolism.^28^ Similarly, genes related to oxidative metabolism and TCA cycle were downregulated in *vegfc*^hy−/−^:*vegfd*^−/−^ mutants, which also occurs following pressure overload in adult mice. There is a strong interplay between myocardial metabolism, activation of inflammatory signaling pathways, expression of cytokines, and immune cell infiltration in heart failure and many of these processes are characteristic of maladaptive hypertrophy of the heart in mammals.^28,56,57^ Mechanisms linking these processes are beginning to be unraveled, but given the strong cross talk between these processes it has been difficult to precisely deconvolute the molecular interplay.^57^ The current study reveals an association between cardiac hypertrophy, metabolic disruption, and inflammation in organisms lacking a functional lymphatic vascular system. Given that VEGF-C/VEGFR3 pathway inhibitors are being developed for cancer, it will be important to consider the impact of these treatments on the cardiovascular system before translation to the clinic.

Our findings reveal a previously unappreciated, context-dependent requirement for the lymphatic vasculature in cardiac growth and regeneration. Cardiac hypertrophy, metabolic disruption, and inflammation are likely to influence the outcome of therapeutic approaches designed to target lymphangiogenesis in ischemic heart disease.

## Methods

### Zebrafish strains

Ethical approval for zebrafish procedures was obtained from The University of Queensland Animal Ethics Committee (AE21513). All zebrafish strains were maintained at The University of Queensland under standard husbandry conditions with a 14 h light, 10 h dark cycles. Transgenic zebrafish lines used in this study included Tg(−5.2lyve1b:DsRed)^nz101^,^29^ Tg(prox1a:KalTA4)^uq3bh^:Tg(10xUAS: Venus),^30^ Tg(kdrl:EGFP)^s834^, vegfd^uq9bh^ and vegfc^hu5055^.^25^ In addition, to label the lymphatic vasculature in vegf mutants, vegfd^uq9bh^;vegfc^hu5055^ double mutants^25^ were outcrossed to Tg(−5.2lyve1b:DsRed).^29^ For mutant genotyping, DNA extraction from fin clips was performed as previously described and genotyping was performed using KASP assays (LGC Genomics). Fish body length was manually measured with a ruler, from the tip of the mouth to the body/caudal fin juncture. All experiments were performed on size-matched fish between 28 and 31 mm in length.

### Cardiac injury in adult zebrafish

Adult fish were anesthetized in aquarium water containing 0.2 mg/ml of Tricaine and secured ventral side up in a slotted sponge. Watchmaker forceps (Dumont #5 91150-20) were used to remove the surface scales and penetrate the skin, muscle, and pericardial sac. Ventricular resection was performed as previously described.^36,37,58^ The ventricle was gently pulled at the apex and 20% of the ventricular apex was cut with Vannas scissors (8 cm, Angled 5 mm, 0.1 mm Tips). Cryoinjury was performed as previously described.^38–40,43^ The silvery epithelial layer of the hypodermis was gently torn with the tip of the scissors to directly access the beating ventricle. The cryoprobe was made with stainless steel, the tip of the applicator was 6 mm long and 0.8 mm in diameter. The cryoprobe was taken out of liquid nitrogen for 10 s before being placed on the ventricle for 24 s, as previously described.^38^ After surgeries, fish were immediately returned to system water. For EdU labeling, fish were anesthetized in water containing 0.2 mg/ml of Tricaine and 10 μl of EdU (5-ethynyl-2′-deoxyuridine from Thermo Fisher) solution (1 μg/μl in PBS) was injected into the abdominal cavity at specified timepoints.

### Fin injury in adult zebrafish

Adult fish were anesthetized in aquarium water containing 0.2 mg/ml of Tricaine. For resection injury, an excision of a small portion of the caudal fin was performed using a scalpel as previously described.^41^ For cryoinjury, the cryoprobe was made with stainless steel, the tip of the applicator was 6 mm long and 0.8 mm in diameter. The cryoprobe was taken out of liquid nitrogen for 10 s before being placed on the caudal fin for 1 min. After surgery, fish were immediately returned to system water.

### Sample preparation for histology

Zebrafish were anesthetized in aquarium water containing 0.6 mg/ml of Tricaine and then euthanized by rapid decapitation. Zebrafish were collected intact and paraformaldehyde-fixed (4% in phosphate buffered saline (PBS; Sigma)) overnight at 4 °C. Hearts were subsequently excised and dissected and the pericardial membrane removed before being placed in paraformaldehyde (4% in PBS) for 1 h at room temperature. Prior to further processing, hearts were washed three times in PBS and DsRed2 and/or Venus reporter expression was monitored in whole hearts before further analyses. For histology, hearts were equilibrated overnight in 15% sucrose in PBS, mounted in Tissue-Tek® O.C.T. compound (ProSciTech), and frozen for cryosectioning.

### Immunohistochemistry

For whole-mount immunostaining, hearts were permeabilized with PBST (PBS + 0.3% Triton X-100 (AMRESCO) for 30 min and incubated with 1% BSA in PBST overnight at 4 °C to block nonspecific binding of the antibodies. Hearts were incubated with the diluted primary antibody in blocking solution (1% BSA in PBST) in a humidified chamber for 1 h at room temperature and overnight at 4 °C. The following primary antibodies were used: chicken anti-GFP (1:200; ABCAM ab13970), rabbit anti-RFP (1:100; ABCAM ab62341), mouse anti-Tropomyosin (1:200; Sigma T9283), and rabbit anti-Fibronectin (1:50; F3648 Sigma). Hearts were washed six times with PBST and incubated with the appropriate secondary fluorescent antibodies (1:400: Goat anti-rabbit/mouse/or chicken Alexa Fluor Thermo Fisher Scientific) in blocking solution in a humidified chamber overnight at 4 °C. Samples were then washed six times in PBST and analyzed. Sagittal sections were obtained (8-μm thickness) at −24 °C using a cryostat. For immunodetection on heart sections, cryosections were dried for 20 min at room temperature, permeabilized with PBSTw (PBS + 0.1% Tween-20 (Sigma)) for 15 min and incubated with 10% Goat Serum (PCN5000, Life Technologies) in PBSTw for 1 h at room temperature to block nonspecific binding of the antibodies. Sections were then incubated with the diluted primary antibody in 5% Goat Serum/PBSTw solution in a humidified chamber overnight at 4 °C. Rabbit anti-fibronectin (1:50; Sigma F3648), rabbit anti-RFP (1:100; ABCAM ab62341) and mouse anti-Tropomyosin (1:200; Sigma T9283) primary antibodies were used. Sections were washed three times with PBSTw and incubated with the appropriate secondary fluorescent antibodies (1:400: Goat anti-rabbit or mouse Alexa Fluor) in 5% Normal Goat Serum (Life technologies, PCN5000)/PBSTw solution in a humidified chamber overnight at 4 °C. Sections were then washed three times in PBSTw and analyzed. EdU labeling was carried out using Click-iT EdU Imaging kit C10338 (Invitrogen), according to the manufacturer’s instructions. For cell death detection, cryosections were dried for 20 min at room temperature and then processed using the In Situ Cell Death Detection Kit (TUNEL kit, 11684795910, Roche), according to the manufacturer’s instructions

### Picrosirius Red/Fast Green, hematoxylin/eosin, and acid fushin orange G (AFOG) staining

For Picrosirius Red/Fast Green staining, sections were postfixed for 30 min in 10% formalin (Sigma), followed by overnight fixation in Bouin’s solution (Sigma), and then extensively washed with tap water. Sections were incubated for 1 h in Picrosirius Red and Fast Green solution: 0.1% direct red 80 (Sigma) and 0.1% fast green FCF (Sigma) in saturated aqueous picric acid (1.2% picric acid in water; Sigma). Sections were quickly washed ten times in distilled water, twice in 70% ethanol, twice in 90% ethanol, then in 100% ethanol (2 × 15 min), and finally in Safesolv solution (Q Path®, VWR International; 2 × 15 min), before being mounted in safemount medium (Q Path®, VWR International). For Hematoxylin and Eosin staining, sections were incubated for 5 min in Hematoxylin solution, washed ten times for 1 min in running tap water, and incubated for 3 min in Eosin solution. Sections were washed three times in distilled water, twice in 70% ethanol, twice in 90% ethanol, then in 100% ethanol (2 × 15 min), and finally in Safesolv solution (Q Path®, VWR International; 2 × 15 min), before being mounted in safemount medium (Q Path®, VWR International). For AFOG staining, an AFOG kit (100 T, Clinisciences) was used, according to the manufacturer’s instructions, before being mounted in safemount medium (Q Path®, VWR International).

### In situ hybridization

The flt4 probe was prepared as previously described.^59^ Cryosections were dried for 20 min at room temperature, permeabilized with PBSTw for 20 min, and incubated for 5 min with proteinase K (5 μg/ml, Sigma 3115887001), sections were then postfixed for 10 min in 4% PFA at room temperature. Sections were pre-hybridized with hybridization buffer (50% formamide, 5× SSC, ARNt 1 mg/ml, Heparin 100 ug/ml, Denhart’s 1×, 0.1% Tween 20, 10% CHAPS, 10 mnol/l EDTA) for 24 h in a humidified chamber at 70 °C. Sections were then hybridized with flt4 probe in hybridization buffer (90 ng/ml) for 24 h in a humidified chamber at 70 °C. Sections were then washed in a humidified chamber at 70 °C with hybridization buffer (100%, 75% in 2× SSC, 50% in 2× SSC and 25% in 2× SSC), followed by 2× SSC and twice with 0.2× SSC. Sections were incubated with Anti-Digoxigenin-AP, Fab fragments (1:200: Sigma 11093274910) in 2% Sheep Serum/BSA (2 mg/ml)/PBSTw solution in a humidified chamber overnight at 4 °C. Sections were washed three times with PBSTw and incubated with BM-Purple (1:50, Sigma11442074001) in staining buffer (100 mM Tris-HCl pH 9.5, 50 mM MgCl2, 100 mM NaCl, 0.1% Tween 20) at room temperature for 6 h. Sections were then washed twice in PBS and analyzed.

### RNA extraction from fresh tissue or from cryosections and cDNA library preparation for RNA-seq

For the transcriptomic analyses presented in Fig. 4 and Supplementary Figs 3 and 4, RNA extraction was performed from fresh tissue using Direct-zol™ RNA Miniprep kit (Zymo/ Integrated Science, R2051), according to the manufacturer’s instructions. For the transcriptomic analyses presented in Fig. 7 and Supplementary Fig. 10, RNA extraction was performed from PFA-fixed (4% PFA) and Tissue-Tek® O.C.T. compound (ProSciTech)-embedded heart cryosections (8 μm). A scalpel was used to collect 12 sections of the ventricle per sample. Sections were transferred to a tube on ice containing 100 μl of TRIzol® Reagent (15596018, Invitrogen) and then processed with Direct-zol™ RNA Microprep kit (Zymo/ Integrated Science, R2061), according to the manufacturer’s instructions. RNA quality was examined with the High Sensitivity RNA ScreenTape on the TapeStation (Agilent) before library preparation. For RNA-seq using fresh tissue, libraries were generated using TruSeq Stranded mRNA Library Prep Kit (#20020595, Illumina) according to the manufacturer’s instructions. For RNA-seq using fixed tissue, ribosomal RNA was depleted from total RNA using NEBNext rRNA Depletion Kit (Human/Mouse/Rat) (E6310, New England Biolabs) followed by library generation using NEBNext Ultra II Directional RNA Library Prep Kit for Illumina (E7760, New England Biolabs) to select library fragment sizes of 200–400 bp, according to the manufacturer’s protocol. Libraries were validated on the TapeStation and sequenced for 150 cycles, 25 million reads, paired-end sequencing on HiSeq4000 for RNA-seq using fixed tissue, or NextSeq550 for RNA-seq using fresh tissue (Illumina).

### RNA-seq and bioinformatic analyses

Paired sequence reads underwent quality trimming using the Skewer (version 0.2.2) with default setting.^60^ For RNA-seq using fresh tissue, sequencing reads were aligned to the zebrafish genome (GRCz11.95) using STAR (version 2.5.2a) with default settings. For RNA-seq using fixed tissue, reads were aligned to the reference genome using Burrows Wheeler Aligner (version 0.7.17) with default settings.^61^ Reads aligning to Ensembl-annotated whole genes on the correct strand with a strict mapping quality (*Q* ≥ 20) were counted with feature Counts and used to construct a data matrix comprising genes with an average of 10 reads or more per sample across the experiment. Heatmaps were generated from normalized and scaled gene expression values using Complex Heatmap package (version 1.18.1) with R in RStudio.^62^ Differential gene expression analysis was performed using edgeR (v3.22.4) with R in RStudio.^63^ GSEA was performed on these data as previously described using publicly available gene sets (www.broadinstitute.org/gsea/msigdb) to determine the enrichment of Reactome pathways.

### Imaging

Imaging of heart slices was performed using a Dragonfly Spinning Disk confocal microscope (ANDOR Oxford Instruments Company). For whole-mount imaging, the heart was mounted in 0.5% low-melting-point agarose and imaged using a Zeiss LSM 710 FCS confocal microscope. Images were processed using ImageJ 1.47 software (National Institute of Health).

### Enzymatic dissociation of cardiomyocytes, staining, and quantification of cardiomyocyte size

Zebrafish ventricles were excised and dissected and the pericardial membrane was removed before being placed in paraformaldehyde (4% in PBS) for 1 h at room temperature, followed by three times washes in PBS. Ventricles were transferred in collagenase solution at 0.5 U/mL in 0.2% NaN3/PBS (Collagenase B 11088807001, Roche) in a tube shaker with oscillation at 1000 rpm at 37 °C. Collection of supernatant was performed every 4 h and the supernatant was transferred to the same volume of 0.2% NaN3/FBS. Digested material was stored at 4 °C between collections. Collection of supernatant was performed every 4 h until the entire ventricle was dissociated. Once dissociation was completed, cardiomyocytes were centrifuged at 1000 × *g* for 3 min. For immunostaining, cardiomyocytes were incubated with 4% BSA, 0.2% Triton X-100, 1 mM EDTA, 0.02% NaN3 in PBS (referred to as blocking buffer) for 10 min at room temperature, then cardiomyocytes were incubated with the anti-Tropomyosin (1:200; Sigma T9283) antibody in blocking buffer for 1 h at room temperature. Cardiomyocytes were washed with blocking buffer and incubated with the appropriate secondary fluorescent antibody (1:400: Goat anti-mouse Alexa Fluor Thermo Fisher Scientific) in blocking buffer for 1 h at room temperature. Cardiomyocytes were then washed in blocking buffer before being mounted in Fluoromount-G (Thermo Fisher Scientific) and analyzed. For quantification of cardiomyocyte volume, width, and length, Z-stack imaging was performed at ×60 magnification for a total of ~1600 cardiomyocytes (~200 per sample, *n* = 8 samples) using a Dragonfly Spinning Disk confocal microscope (ANDOR Oxford Instruments Company). All images were similarly processed using ImageJ 1.47 software (National Institute of Health). Automated quantification of cardiomyocyte volume was performed using ImageJ Trainable Weka Segmentation macro.^64^ Cardiomyocyte width and length were quantified manually using ImageJ measurement tools. The code can be accessed via GitHub using the following link (https://github.com/Jacob-Smith-MCRI/Cardiomyocyte-Volume-Calculator).

### Quantification of ventricle size, myocardial volume, collagen deposition, interstitial volume, cell death, and injury size

Heart sections labeled with Picrosirius Red/Fast Green were used to analyze ventricle volume, myocardium volume and the presence of collagen and non-collagenous proteins. Whole hearts were sectioned (sagittal sections of 8 μm) and Picrosirius Red/Fast Green labeling was performed at 56 μm intervals. Images were captured for each labeled section. All images were similarly processed using ImageJ 1.47 software (National Institute of Health). For ventricle size and myocardium volume measurements, selections were carefully traced around the entire ventricle and around the myocardial wall. Selected surface areas were then quantified using ImageJ software. Surface areas were then summed giving the total ventricle volume and myocardial volume for each sample. Myocardial volume was then normalized to ventricle volume. For interstitial volume measurements, color thresholds were adjusted to select the area corresponding to the interstitial space and interstitial volumes were measured and normalized to ventricular volumes. For quantification of collagen fibers, manual selections were captured within the ventricle on RGB images and color thresholds were adjusted to only select the area corresponding to collagen fiber staining (dark pink); four sections were processed for each sample. Collagen surface areas were measured and normalized to ventricular surface areas. Transverse heart sections of 8 μm labeled with Acid Fushin Orange G were used to quantify infarct size. Color thresholds were adjusted to dark orange to select the area corresponding to the damaged tissue and damaged tissue volumes were measured (eight sections were processed for each sample). Selected surface areas of transverse heart sections labeled with TUNEL (11684795910, Roche) were quantified using ImageJ software. Surface areas were then summed giving the total cell death volume for each sample (six sections were processed for each sample).

### Quantification of cardiomyocyte number and proliferation

Heart sections co-labeled with Tropomyosin, EdU, and Dapi were used to analyze cardiomyocyte proliferation or number. Whole hearts were sectioned (sagittal sections of 8 μm) and EdU, Dapi, and Tropomyosin co-labeling were performed at 56 μm intervals. For quantification of cardiomyocyte number, five sections were processed for each sample. Dapi positive/Tropomyosin positive nuclei were manually counted in a surface selection of the ventricle and normalized to total surface area (1000 μm^2^). For cardiomyocyte proliferation analysis, three sections corresponding to the injured area were processed for each sample. EdU positive/Tropomyosin positive nuclei were manually counted in a surface selection at the apex of the ventricle and normalized to the tropomyosin positive surface selection area.

### Statistical analysis

The nonparametric Mann–Whitney test was used for comparison of two means and linear regression analysis was performed to analyze correlations using Prism software (GraphPad). Kolmogorov-Smirnov tests was used for comparison of two frequency distributions using Prism software (GraphPad). Comparison of means are presented as the mean ± standard error of the mean (sem). Correlation analyses are presented as XY dot plots with the slope, *R* square, and *P*-value. For all analyses *P* < 0.05 was considered statistically significant.

### Reporting summary

Further information on research design is available in the Nature Research Reporting Summary linked to this article.

## Supplementary information

Supplementary Files.

Supplementary Data Set 1.

Supplementary Data Set 2.

Supplementary Data Set 3.

Supplementary Data Set 4.

Reporting Summary Checklist

## Data Availability

All data sets associated with the current study are available from the corresponding author on reasonable request. RNA-seq data have been deposited at the Gene Expression Omnibus under the accession number GSE133131.^65^

## Supplementary information

**Supplementary Information** accompanies the paper on the *npj Regenerative Medicine* website (10.1038/s41536-019-0079-2).

## Code availability

Automated quantification of cardiomyocyte volume was performed using ImageJ 1.47 software (National Institute of Health) with ImageJ Trainable Weka Segmentation macro.^64^ The code can be accessed via GitHub using the following link (https://github.com/Jacob-Smith-MCRI/Cardiomyocyte-Volume-Calculator).
