# Supplementary material for: Vegfc/d-dependent regulation of the lymphatic vasculature during cardiac regeneration is influenced by injury context
Source: NPJ Regen Med. 2019 Aug 22;4:18. doi: 10.1038/s41536-019-0079-2 (PMC6706389; doi:10.1038/s41536-019-0079-2)
Supplement: Supplementary file 1 — Supplementary Files. [file 41536_2019_79_MOESM1_ESM.pdf]

Supplementary Files

Supplementary Figure 1: Epicardial and myocardial blood vessels are detectable in *vegfc*$^{+/+}$*:vegfd*$^{+/+}$ control hearts and *vegfc*$^{hy-/-}$ *vegfd*$^{-/-}$mutant hearts.

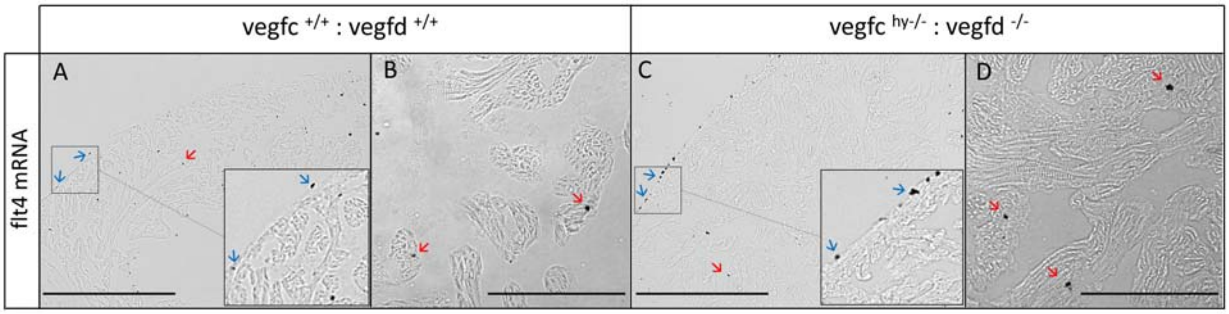

A-D: *flt*4 in situ hybridization was performed on **vegfc**$^{+/+}$**:vegfd**$^{+/+}$ (A and B) and **vegfc**$^{hy-/-}$**:vegfd**$^{-/-}$ (C and D) mutant heart sections. Blue arrows denote blood vessels (expressing *flt*4 mRNA) on the epicardial surface of the heart (A and C) and red arrows denote blood vessels within the myocardium (B and D). Scale bar length 500µm (A and C) or 100µm (D and E).

Supplementary Figure 2: Characterization of collagen fibers, non-collagenous content, proliferation and cardiomyocyte isolation in the *vegfc*$^{hy-/-}$:*vegfd*$^{-/-}$ hearts.

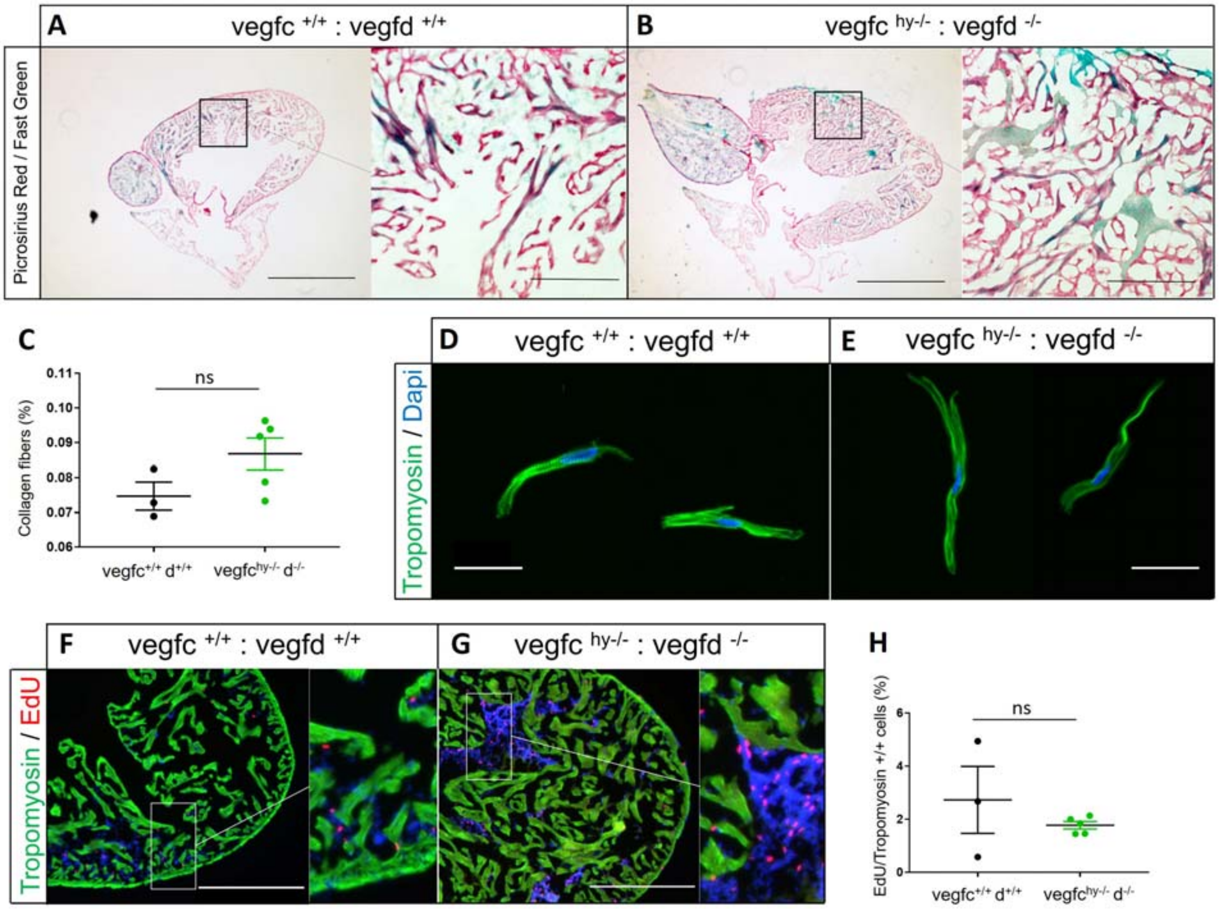

A and B: Picrosirius red and Fast Green staining was performed on vegfc$^{+/+}$:vegfd$^{+/+}$ and vegfc$^{-/-}$:vegfd$^{hy-/-}$ hearts. Pink staining marks muscle fibers, bright pink staining represents collagen fibers and green staining represents non-collagenous proteins. Note the abundance of non-collagenous proteins in the *vegfc*$^{hy-/-}$:*vegfd*$^{-/-}$ hearts within the interstitial space. Scale bar length 0.8mm or 160µm for the magnification. **C:** Quantification of collagen fibers in *vegfc*$^{+/+}$*:vegfd*$^{+/+}$ and *vegfc*$^{hy-/-}$*:vegfd*$^{-/-}$ mutant hearts. There was no statistically significant difference in fibrosis between control and mutant hearts. Statistical analysis: unpaired, nonparametric, Mann-Whitney test was used to compare two means. Data are presented as the mean ± standard error of the mean (sem). **D and E:** Enzymatic digestion was performed on *vegfc*$^{+/+}$*:vegfd*$^{+/+}$ or *vegfc*$^{hy-/-}$*:vegfd*$^{-/-}$ mutant ventricles and tropomyosin and Dapi staining were

performed on isolated cardiomyocytes. Scale bar length 30µm. **F-H:** EdU was injected in *vegfc*$^{+/+}$:*vegfd*$^{+/+}$ and *vegfc*$^{hy-/-}$:*vegfd*$^{-/-}$ fish every 7 days for 28 days to monitor proliferation in cardiac cells. Tropomyosin and EdU staining was performed on *vegfc*$^{+/+}$*:vegfd*$^{+/+}$ (F) and vegfc$^{hy-/-}$:vegfd$^{-/-}$ hearts (G) and cardiomyocyte proliferation was quantified (H). Scale bar length 0.3mm. Cardiomyocyte proliferation occurs at a similar level in *vegfc*$^{+/+}$:*vegfd*$^{+/+}$ and *vegfc*$^{hy-/-}$:*vegfd*$^{-/-}$ hearts. Statistical analysis: unpaired, nonparametric, Mann-Whitney test was used to compare two means. Data are presented as the mean ± standard error of the mean (sem).

Supplementary Figure 3: V*egfc*$^{hy-/-}$;*vegfd*$^{-/-}$ mutant zebrafish hearts share common transcriptional programs with pathological cardiac hypertrophy in mice.

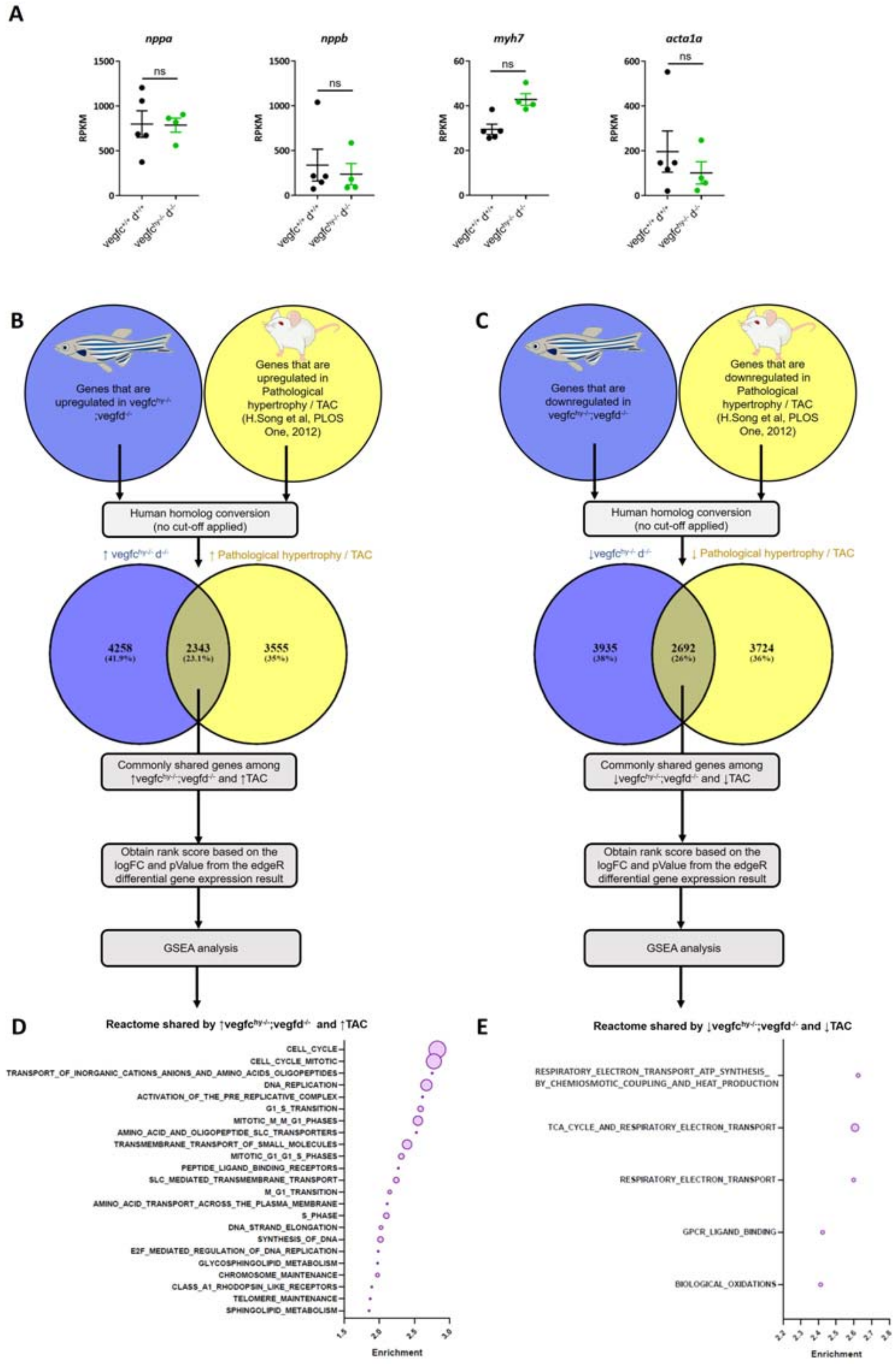

A: Classic cardiac hypertrophy stress markers are not up-regulated in v*egfc*$^{hy-/-}$;*vegfd*$^{-/-}$ mutant ventricles. RNA-seq transcript levels for each gene presented as reads per kilobase million (RPKM). Data presented as mean ± standard error of the mean (sem). **B-C:** Flow diagrams summarizing bioinformatic pipeline used to compare up- and down-regulated transcripts in v*egfc*$^{hy-/-}$;*vegfd*$^{-/-}$ zebrafish mutants to published data for TAC-induced pathological cardiac hypertrophy in mice. All up-regulated (B) or down-regulated (C) genes in v*egfc*$^{hy-/-}$;*vegfd*$^{-/-}$ mutants or all up-regulated (B) or down-regulated (C) genes in the TAC-induced pathological hypertrophy model were cross-referenced to pull out shared genes. **D:** GSEA of shared up-regulated transcripts between *vegfc*$^{hy-/-}$;*vegfd*$^{-/-}$ mutant ventricles and TAC-induced pathological hypertrophy program. **E:** GSEA of shared down-regulated transcripts between *vegfc*$^{hy-/-}$;*vegfd*$^{-/-}$ mutant ventricles and TAC-induced pathological hypertrophy program. Representative pathways are highlighted with circles and gene set sizes are represented by circle sizes.

Supplementary Figure 4: V*egfc*$^{hy-/-}$;*vegfd*$^{-/-}$ mutant zebrafish hearts share common transcriptional programs with physiological cardiac hypertrophy in mice. .

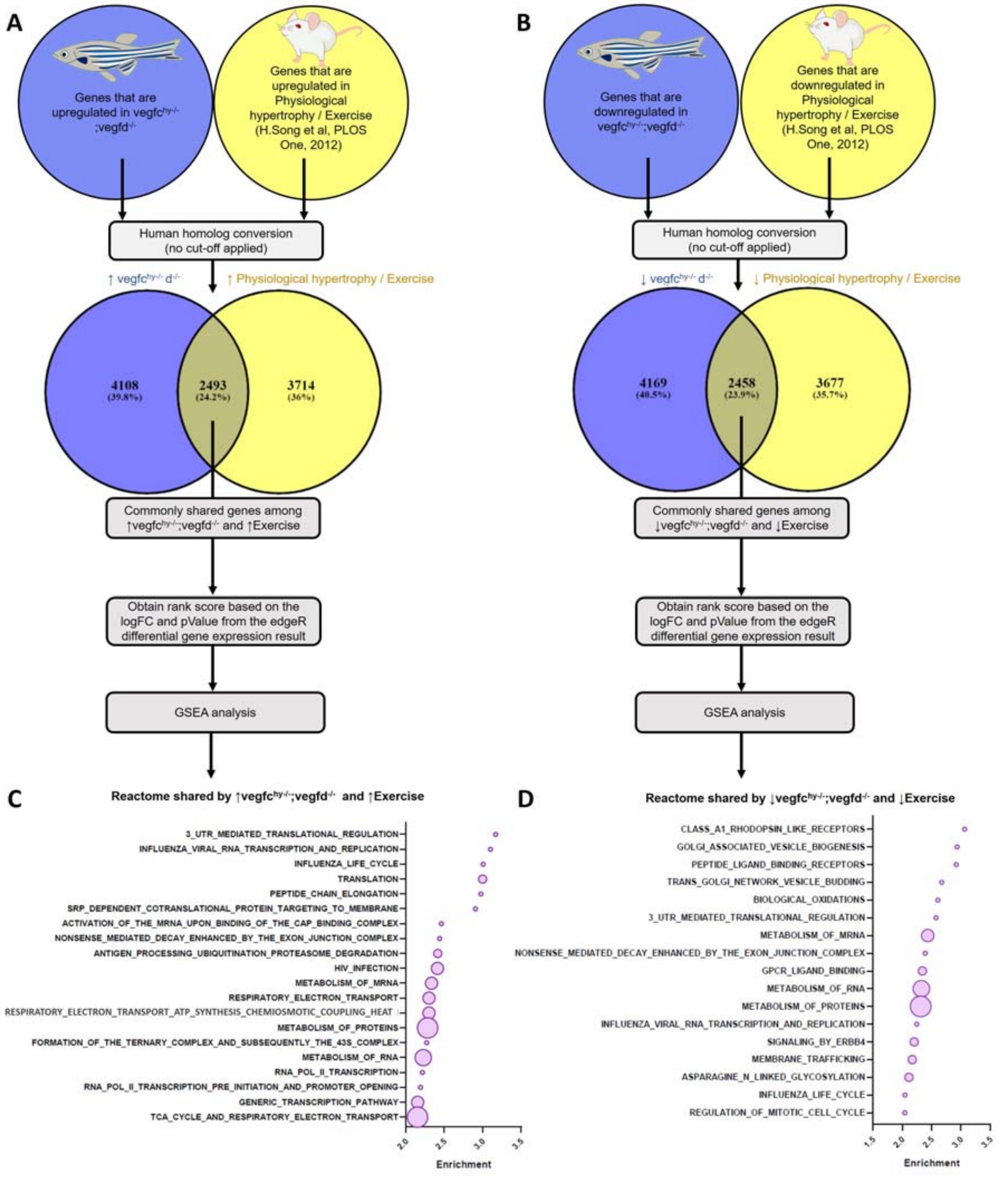

A-B: Flow diagrams summarizing bioinformatic pipeline used to compare up- and down-regulated transcripts in v*egfc*$^{hy-/-}$;*vegfd*$^{-/-}$ zebrafish mutants to published data for exercise-induced physiological cardiac hypertrophy in mice. All up-regulated (B) or down-regulated (C) genes in v*egfc*$^{hy-/-}$;*vegfd*$^{-/-}$ mutants or all up-regulated (B) or down-regulated (C) genes in the exercise-induced physiological hypertrophy model were cross-referenced to pull out shared genes. **D:** GSEA of shared up-regulated transcripts between *vegfc*$^{hy-/-}$;*vegfd*$^{-/-}$ mutant ventricles and exercise-induced physiological hypertrophy program. **E:** GSEA of shared down-regulated transcripts between *vegfc*$^{hy-/-}$;*vegfd*$^{-/-}$ mutant ventricles and exercise-induced physiological hypertrophy program. Representative pathways are highlighted with circles and gene set sizes are represented by circle sizes.

Supplementary Figure 5: Cryoinjury stimulates a lymphangiogenic response in the caudal fin.

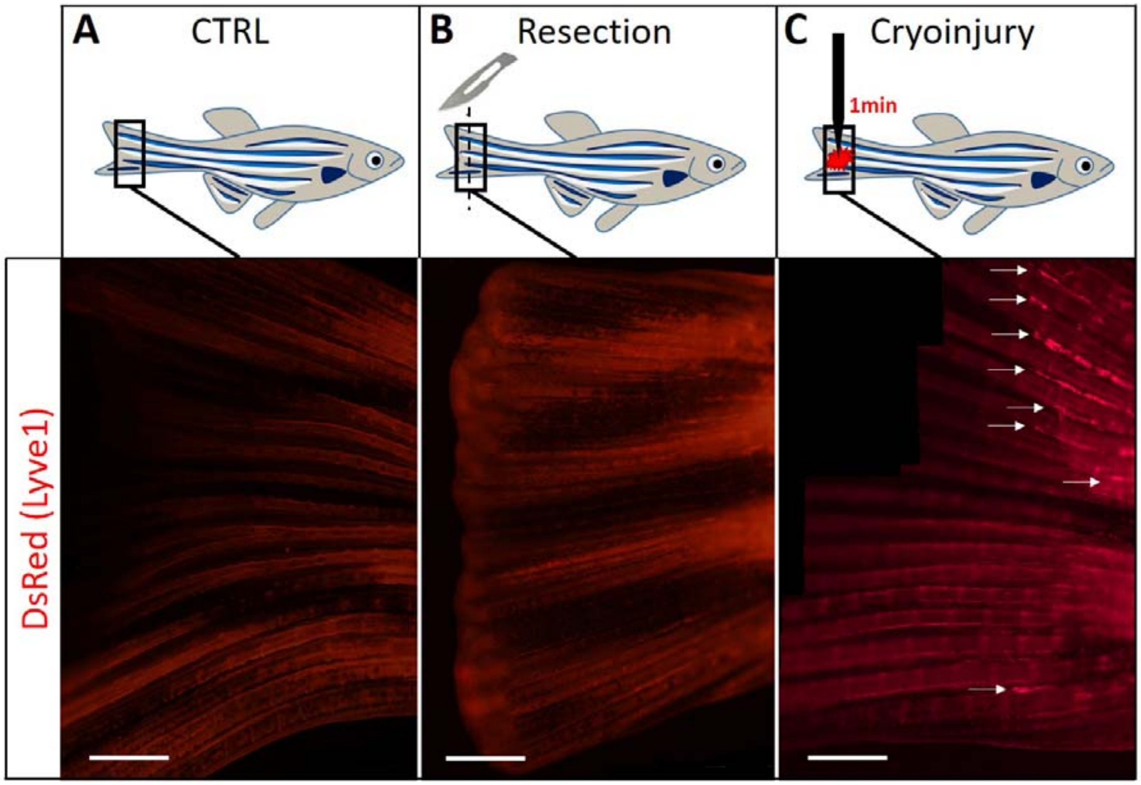

A: In control fish (without injury), dsRed (Lyve1) is not detected in the caudal fin indicating that lymphatic vessels are not present in the control zebrafish fin. **B:** The caudal fin was resected (along the dotted line) and observed 4 days post resection. DsRed (lyve1) is not detected in the zebrafish fin 4 days post amputation indicating that resection injury does not induce a lymphangiogenic response in the fin. **C:** The caudal fin was cryoinjured for 1 minute and observed 4 days post cryoinjury. A robust lymphangiogenic response to cryoinjury in the caudal fin was evident by the presence of DsRed (Lyve1) positive vessels between the fin rays (white arrows). Scale bar length 1mm.

Supplementary Figure 6: Accumulation of dead cells in a subset of vegfc$^{hy-/-}$ vegfd$^{-/-}$ mutant hearts following cryoinjury.

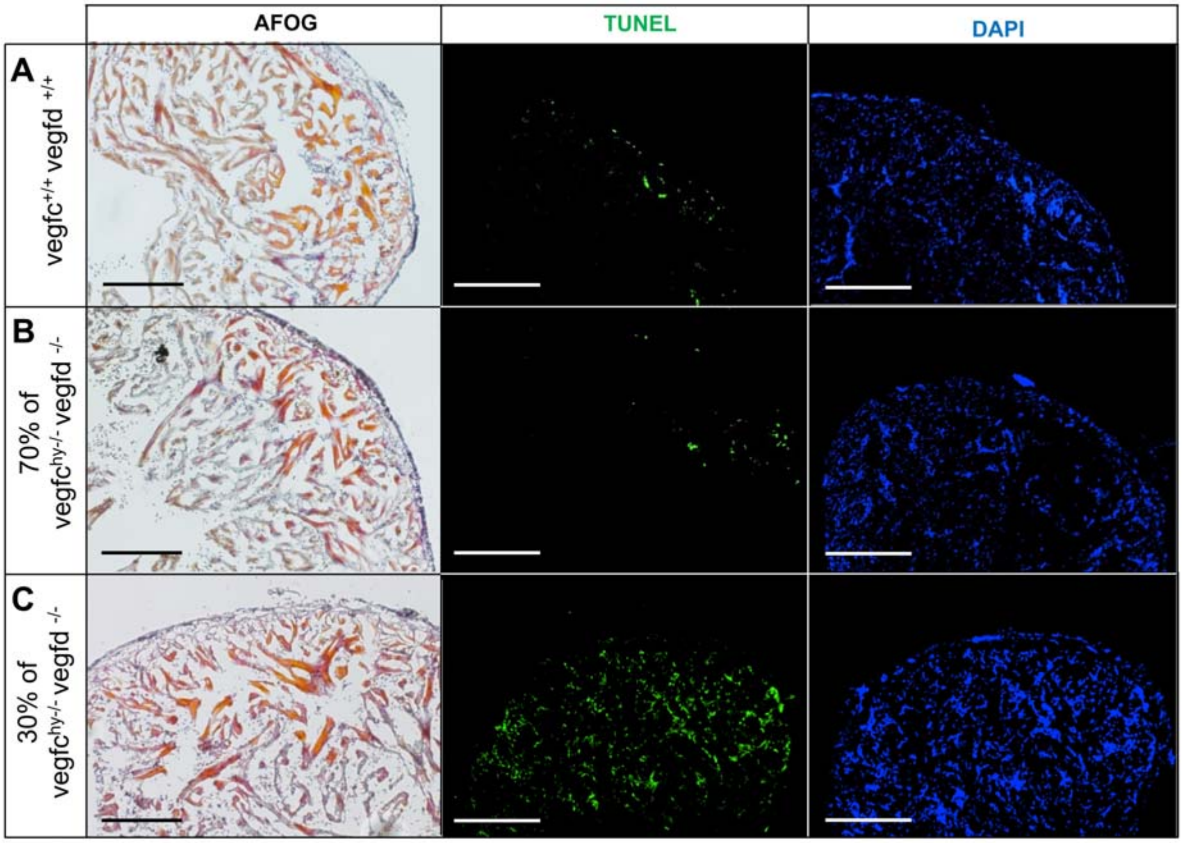

A-C: First column: Acid Fucshin Orange G (AFOG) staining. Dark orange staining indicates damaged tissue. Note that injury size is similar between control and mutant hearts. Scale bar length 100µm. Second and third columns: TUNEL (second column) and DAPI (third column) staining were performed on vegfc$^{+/+}$:vegfd$^{+/+}$ and vegfc$^{-/-}$:vegfd$^{hy-/-}$ hearts at 1 day post cryoinjury. Note the abundance of dead cells in 3/8 of the *vegfc*$^{hy-/-}$:*vegfd*$^{-/-}$ hearts. Scale bar length 200µm.

Supplementary Figure 7: Lymphatic vessels are not observed in the ventricle or at the site of injury in vegfc$^{hy-/-}$ vegfd$^{-/-}$mutant hearts after cryoinjury.

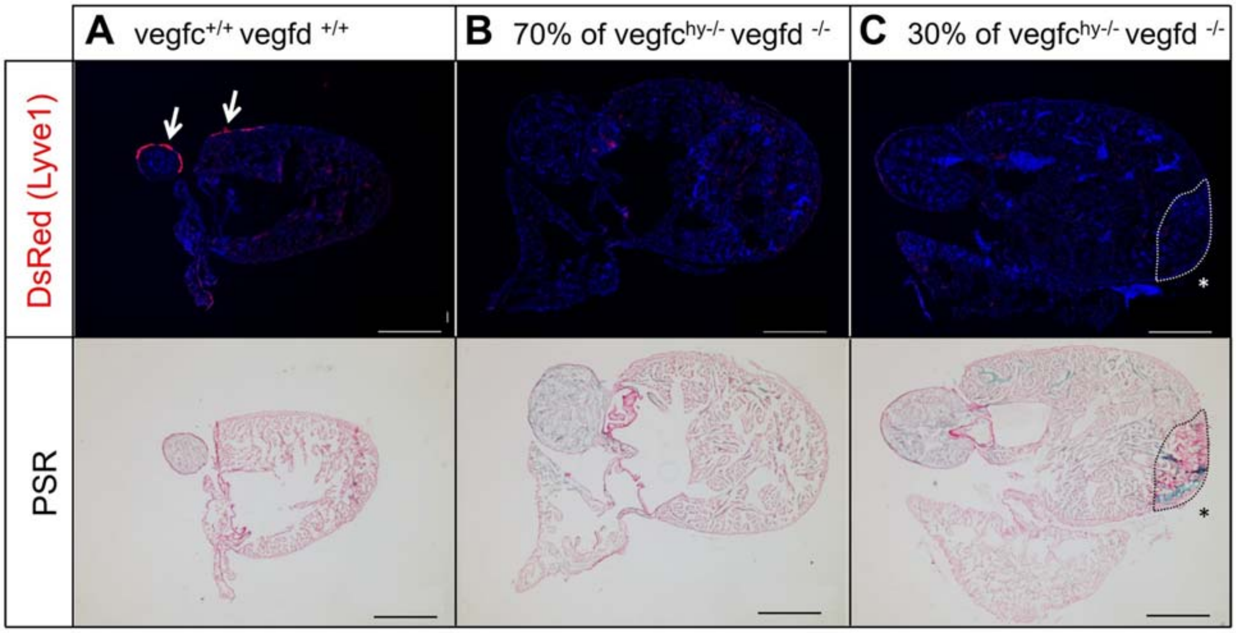

A-C: DsRed or PSR staining was performed on *vegfc*$^{+/+}$*:vegfd*$^{+/+}$:Tg(-5.2lyve1b:Dred) and vegfc$^{hy-/-}$:vegfd$^{-/-}$:Tg(-5.2lyve1b:Dred) mutant lines 6 months after cryoinjury. Arrows denote lymphatic vessels (DsRed staining) on the epicardial surface of the heart. Note absence of lymphatic vascular network in the ventricle and bulbus arteriosus of *vegfc*$^{hy-/-}$;*vegfd*$^{-/-}$ double mutants. Note absence of lymphatic vessels at the site of injury in vegfc$^{hy-/-}$;vegfd$^{-/-}$ double mutants (*). Scale bar length 0.5mm.

Supplementary Figure 8: Cardiomyocyte proliferation is not correlated with regeneration or myocardial volume in control and *vegfc*$^{hy-/-}$:*vegfd*$^{-/-}$ hearts.

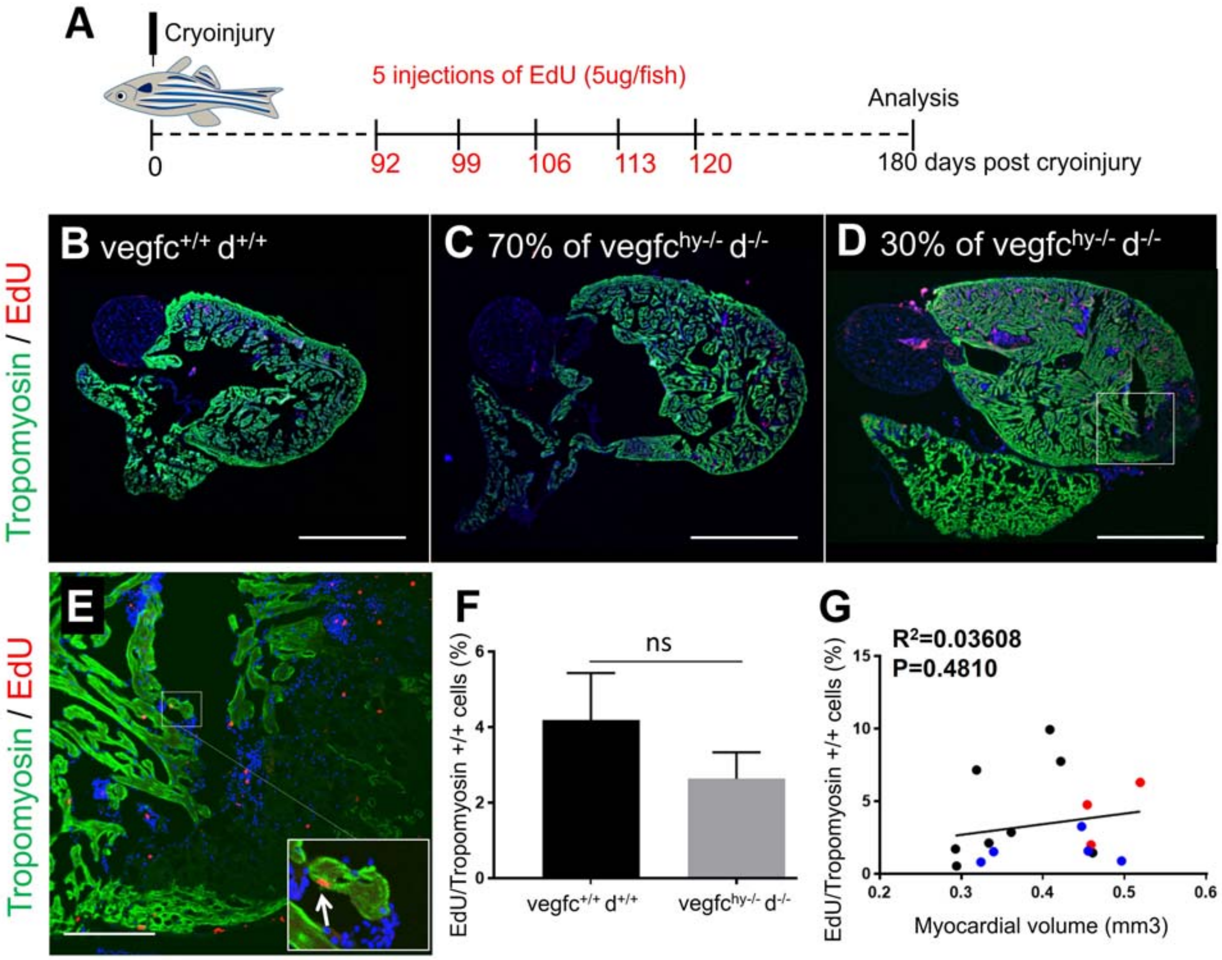

A: EdU was injected in *vegfc*$^{+/+}$:*vegfd*$^{+/+}$ and *vegfc*$^{hy-/-}$:*vegfd*$^{-/-}$ fish to monitor proliferation in cardiac cells after cryoinjury. Scale bar length 0.8mm. **B-D:** Tropomyosin and EdU staining was performed on *vegfc*$^{+/+}$*:vegfd*$^{+/+}$ and vegfc$^{hy-/-}$:vegfd$^{-/-}$ hearts 180 days after cryoinjury. Scale bar length 0.8mm. **E:** Magnification of white box in D. Scale bar length 150µm. Note that cardiomyocyte proliferation still occurs in *vegfc*$^{hy-/-}$:*vegfd*$^{-/-}$ mutants that had impaired regenerative capacity. **F:** Cardiomyocyte proliferation occurs at a similar level in *vegfc*$^{+/+}$:*vegfd*$^{+/+}$ and *vegfc*$^{hy-/-}$:*vegfd*$^{-/-}$ hearts. **G:** Correlation analyses between myocardial volume and cardiomyocyte proliferation in *vegfc*$^{+/+}$*:vegfd*$^{+/+}$ and *vegfc*$^{hy-/-}$

:vegfd$^{-/-}$ mutant hearts 6 months after cryoinjury. Black plots represent *vegfc*$^{+/+}$*:vegfd*$^{+/+}$ hearts, blue plots represent *vegfc*$^{hy-/-}$*:vegfd*$^{-/-}$ hearts that showed complete heart regeneration, red plots represent *vegfc*$^{hy-/-}$ *:vegfd*$^{-/-}$ hearts that had impaired cardiac regenerative capacity. Statistical analysis: unpaired, nonparametric, Mann-Whitney tests was used to compare two means. Data are presented as the mean ± standard error of the mean (sem). Correlation analyses are presented as a dot plot with the slope, R square and P value. n= 8 *vegfc*$^{hy-/-}$:*vegfd*$^{-/-}$, n=8 *vegfc*$^{+/+}$:*vegfd*$^{+/+}$.

Supplementary Figure 9: Cardiomyocyte proliferation is not correlated with infarct size at day 19 following cryoinjury in control and *vegfc*$^{hy-/-}$:*vegfd*$^{-/-}$ hearts.

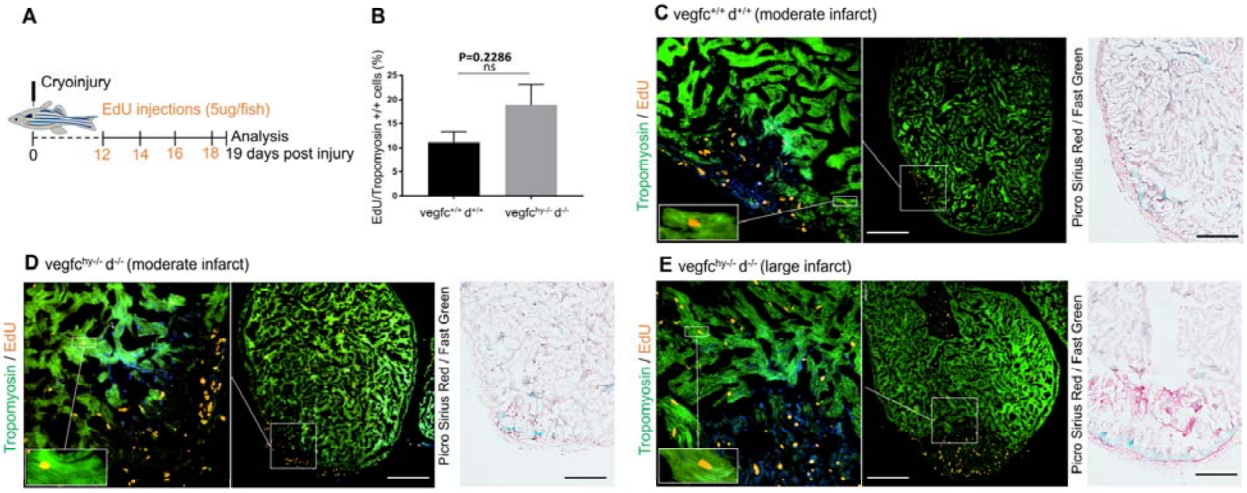

A: EdU was injected in *vegfc*$^{+/+}$:*vegfd*$^{+/+}$ and *vegfc*$^{hy-/-}$:*vegfd*$^{-/-}$ fish to monitor proliferation in cardiac cells after cryoinjury. **B:** Cardiomyocyte proliferation occurs at a similar level in *vegfc*$^{+/+}$:*vegfd*$^{+/+}$ and *vegfc*$^{hy-/-}$:*vegfd*$^{-/-}$ hearts 19 days after cryoinjury. **C-E:** Tropomyosin/EdU and Picro Sirius red/Fast green staining were performed on *vegfc*$^{+/+}$:*vegfd*$^{+/+}$ and vegfc$^{hy-/-}$:vegfd$^{-/-}$ hearts on day 19 after cryoinjury. Statistical analysis: unpaired, nonparametric, Mann-Whitney test was used to compare two means. Data are presented as the mean ± standard error of the mean (sem). Correlation analyses are presented as a dot plot with the slope, R square and P value. n= 4 *vegfc*$^{hy-/-}$:*vegfd*$^{-/-}$, n=3 *vegfc*$^{+/+}$:*vegfd*$^{+/+}$. Scale bar length 245µm (Tropomyosin/Edu) or 200µm (Picro Sirius red/Fast green).

Supplementary Figure 10: Transcriptional programs induced by cryoinjury in v*egfc*$^{hy-/-}$;*vegfd*$^{-/-}$ mutant hearts.

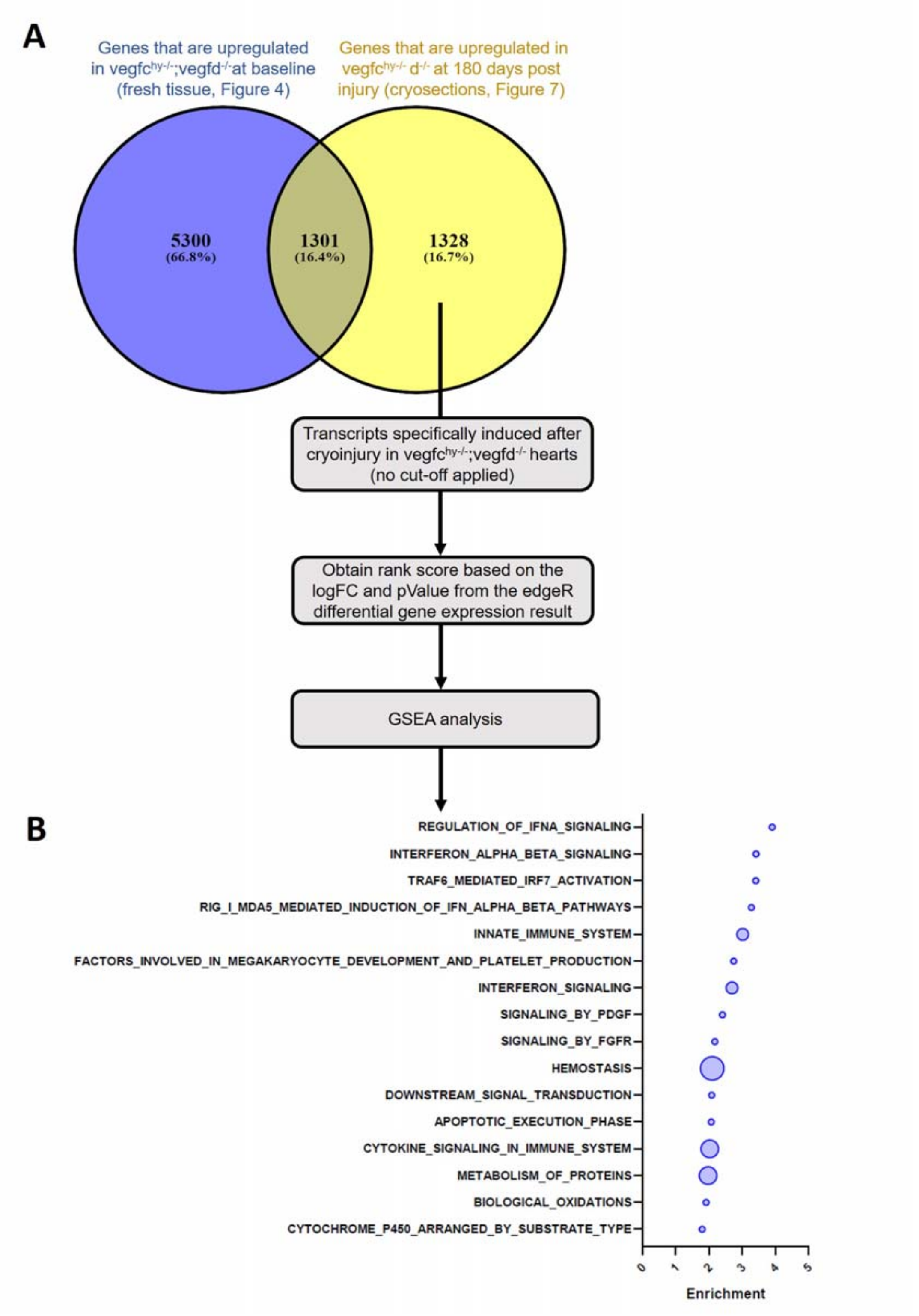

A: Flow diagram summarizing bioinformatic pipeline used to compare the v*egfc*$^{hy-/-}$;*vegfd*$^{-/-}$ baseline transcriptome derived from fresh whole ventricle tissue (Figure 4) to the v*egfc*$^{hy-/-}$;*vegfd*$^{-/-}$ transcriptome derived from cryosections at 180 days post cryoinjury (Figure 7). All up-regulated genes in v*egfc*$^{hy-/-}$;*vegfd*$^{-/-}$ mutants without injury or all up-regulated genes in v*egfc*$^{hy-/-}$;*vegfd*$^{-/-}$ mutants at 180 days post injury were cross-referenced to identify a subset of genes specifically induced by cryoinjury. **B:** GSEA of the specific injury-induced transcripts in *vegfc*$^{hy-/-}$;*vegfd*$^{-/-}$ mutant ventricles after cryoinjury. Representative pathways are highlighted with circles and gene set sizes are represented by circle sizes.

Supplementary Data Set 1: Cardiomyocyte width, length and volume measurements.

Table summarizing width, length and volume measurements performed on *vegfc*$^{+/+}$*:vegfd*$^{+/+}$ or *vegfc*$^{hy-/-}$ *:vegfd*$^{-/-}$ isolated cardiomyocytes. Measurements were performed from a total of ~200 cardiomyocytes per sample (~800 cardiomyocytes per group) comprising n=4 *vegfc*$^{hy-/-}$:*vegfd*$^{-/-}$ ventricles and n=4 *vegfc*$^{+/+}$:*vegfd*$^{+/+}$ ventricles, respectively.

Supplementary Data Set 2: RNA-seq data of cardiac ventricles from *vegfc*$^{+/+}$*:vegfd*$^{+/+}$ controls and *vegfc*$^{hy-/-}$*;vegfd*$^{-/-}$ mutants.

List of differentially regulated genes, their associated Reactome pathways, their fold changes and p-values from the RNA-seq analysis performed on cardiac ventricles from control and mutant zebrafish ventricles at 5 months of age.

Supplementary Data Set 3: RNA-seq data obtained from cryosections of cardiac ventricles from *vegfc*$^{+/+}$*:vegfd*$^{+/+}$ controls and *vegfc*$^{hy-/-}$;*vegfd*$^{-/-}$ mutants at 180 days following cryoinjury.

List of differentially regulated genes, their associated Reactome pathways, their fold changes and p-values from the RNA-seq analysis performed on cryosections from control and mutant ventricles at 180 days following cryoinjury.

Supplementary Data Set 4: RNA-seq data obtained from cryosections of *vegfc*$^{hy-/-}$;*vegfd*$^{-/-}$ mutant hearts that did not regenerate or mutant hearts that recovered completely at 180 days post cryoinjury.

List of differentially regulated genes, their associated Reactome pathways, their fold changes and p-values from the RNA-seq analysis performed on cryosections of *vegfc*$^{hy-/-}$;*vegfd*$^{-/-}$ mutant hearts that did not regenerate (n=3) or on cryosections of mutant hearts which recovered completely (n=5) at 180 days post cryoinjury (presented in Figure 6G-L).
